# Adaptation and validation of the ACMG/AMP variant classification framework for *MYH7*-associated inherited cardiomyopathies: recommendations by ClinGen’s Inherited Cardiomyopathy Expert Panel

**DOI:** 10.1038/gim.2017.218

**Published:** 2018-01-04

**Authors:** Melissa A Kelly, Colleen Caleshu, Ana Morales, Jillian Buchan, Zena Wolf, Steven M Harrison, Stuart Cook, Mitchell W Dillon, John Garcia, Eden Haverfield, Jan D H Jongbloed, Daniela Macaya, Arjun Manrai, Kate Orland, Gabriele Richard, Katherine Spoonamore, Matthew Thomas, Kate Thomson, Lisa M Vincent, Roddy Walsh, Hugh Watkins, Nicola Whiffin, Jodie Ingles, J Peter van Tintelen, Christopher Semsarian, James S Ware, Ray Hershberger, Birgit Funke, for theClinGen Cardiovascular Clinical Domain Working Group

**Affiliations:** 1 Laboratory for Molecular Medicine, Partners Healthcare Personalized Medicine, Boston, Massachusetts, USA; 2 Stanford Center for Inherited Cardiovascular Disease, Stanford University, Stanford, California, USA; 3 Division of Human Genetics, Department of Internal Medicine, Ohio State University, Columbus, Ohio, USA; 4 National Heart and Lung Institute, Imperial College London, London, UK; 5 Invitae Inc., San Francisco, California, USA; 6 Department of Genetics, University of Groningen, University Medical Center Groningen, Groningen, Netherlands; 7 GeneDx Laboratories, Gaithersburg, Maryland, USA; 8 Harvard School of Public Health, Boston, Massachusetts, USA; 9 Clinical Science Center, University of Wisconsin, Madison, Wisconsin, USA; 10 Krannert Institute of Cardiology, Indiana University, Indianapolis, Indiana, USA; 11 Division of Genetics, Department of Pediatrics, University of Virginia, Charlottesville, Virginia, USA; 12 Oxford Medical Genetics Laboratory, Oxford University Hospitals NHS Foundation Trust, The Churchill Hospital, Oxford, UK; 13 Radcliffe Department of Medicine, University of Oxford, Oxford, UK; 14 Royal Brompton & Harefield Hospitals NHS Trust, London, UK; 15 Agnes Ginges Centre for Molecular Cardiology, Centenary Institute and University of Sydney, Sydney, Australia; 16 Department of Clinical Genetics, Academic Medical Center, University of Amsterdam, Amsterdam, Netherlands; 17 Department of Pathology, Massachusetts General Hospital, Boston, Massachusetts, USA; 18 Harvard Medical School, Boston, Massachusetts, USA

**Keywords:** cardiomyopathy, ClinGen, HCM, myosin heavy chain 7, variant interpretation

## Abstract

**Purpose:**

Integrating genomic sequencing in clinical care requires standardization of variant interpretation practices. The Clinical Genome Resource has established expert panels to adapt the American College of Medical Genetics and Genomics/Association for Molecular Pathology classification framework for specific genes and diseases. The Cardiomyopathy Expert Panel selected *MYH7*, a key contributor to inherited cardiomyopathies, as a pilot gene to develop a broadly applicable approach.

**Methods:**

Expert revisions were tested with 60 variants using a structured double review by pairs of clinical and diagnostic laboratory experts. Final consensus rules were established via iterative discussions.

**Results:**

Adjustments represented disease-/gene-informed specifications (12) or strength adjustments of existing rules (5). Nine rules were deemed not applicable. Key specifications included quantitative frameworks for minor allele frequency thresholds, the use of segregation data, and a semiquantitative approach to counting multiple independent variant occurrences where fully controlled case-control studies are lacking. Initial inter-expert classification concordance was 93%. Internal data from participating diagnostic laboratories changed the classification of 20% of the variants (*n* = 12), highlighting the critical importance of data sharing.

**Conclusion:**

These adapted rules provide increased specificity for use in *MYH7*-associated disorders in combination with expert review and clinical judgment and serve as a stepping stone for genes and disorders with similar genetic and clinical characteristics.

## Introduction

Determining the clinical significance of sequence variants is a complex process that involves gathering and assigning relative weights to a multitude of data gathered from a diverse set of resources. Variant classification has evolved in a decentralized fashion leading to a multitude of approaches, most developed by molecular diagnostic laboratories for internal use.

As sequencing tests become a routine tool in managing health, it is vital to harmonize and centralize knowledge and approaches. Key advancements included the creation of the National Center for Biotechnology Information’s ClinVar database (http://www.ncbi.nlm.nih.gov/clinvar/), which has quickly become a valuable centralized resource for clinically classified variants, and the National Human Genome Research Institute–funded Clinical Genome Resource (ClinGen, http://www.clinicalgenome.org), which serves as a body for managing and centralizing clinically relevant genomic knowledge. The historic lack of standardization is a major contributor for interpretation differences, which have been revealed by increased data sharing through ClinVar.^1, 2, 3^ Many of these differences represent misclassifications, which can have serious consequences, especially for medically actionable variants as illustrated by Gaba et al.,^4^ who reported that implantable cardioverter-defibrillators were implanted based on the incorrect classification of a variant as causative for long QT syndrome.

The American College of Medical Genetics and Genomics (ACMG) and the Association for Molecular Pathology (AMP) have responded to the urgent need for an updated variant classification framework by releasing a landmark guidance document,^5^ which has since been adopted by many US and international laboratories.^6^ However, this framework was designed to have universal applicability, and therefore requires significant expertise to be applied correctly for specific diseases and genes.

Lack of clinical domain expert knowledge is emerging as a key contributor to incorrect classifications.^2, 7^ Besides adding disease and gene specifications for certain rules (including flagging rules that are not applicable), there is a critical need to provide more granularity surrounding frequency thresholds that drive the benign spectrum of rules, to specify thresholds for assigning increasing weight depending on the degree of segregation with disease, and to provide guidance surrounding the use of functional data.^7^ Some studies have proposed approaches and solutions;^8, 9, 10, 11^ however, systematic expert-led efforts are needed to create disease- and/or gene-specific derivatives of the original ACMG/AMP framework. ClinGen has emerged as a critical facilitator in this domain and has established a rich infrastructure including disease-specific expert working groups that have been charged with accomplishing this goal.^3^

Here we report the work of ClinGen’s Inherited Cardiomyopathy Expert Panel (CMP-EP), which adapted the ACMG/AMP framework for use in myosin heavy chain 7 (*MYH7*)-associated cardiomyopathies with the aim of improving consistency for variant interpretation and expert curation of reported *MYH7* variants for submission to ClinVar (3 star). These disorders include hypertrophic, dilated, and restrictive cardiomyopathy (HCM, DCM, and RCM), which are collectively among the most prevalent Mendelian conditions and affect 1 in 200–500 individuals.^12, 13^ *MYH7* is the second most common inherited cause of HCM and third most common inherited cause of DCM, primarily due to missense variants that are dominantly inherited, although de novo variants have been reported.^14, 15^

## Materials and methods

### ClinGen’s Inherited Cardiomyopathy Expert Panel

The CMP-EP operates under the umbrella of the Cardiovascular Domain Working Group. Members were selected to provide a balanced representation of expertise in clinical cardiology, clinical research, molecular diagnostics, genetic counseling, and genomic medicine. Additional emphasis was placed on global representation (United States, United Kingdom, the Netherlands, and Australia) to lay the foundation for international harmonization.

### Decision-making framework

A subset of the CMP-EP formed a core task team with clinical as well as molecular diagnostic expertise and representation from three institutions (M.A.K., Partners HealthCare Laboratory for Molecular Medicine; C.C., Stanford University; A.M., Ohio State University). This task team reviewed the original ACMG/AMP framework^5^ and developed proposed changes to adapt them for *MYH7*. Proposed adaptations were discussed by the CMP-EP using conference calls, e-mail, or electronic surveys to arrive at consensus decisions (Supplementary Text S1 online).

### Project design

**Figure 1:**
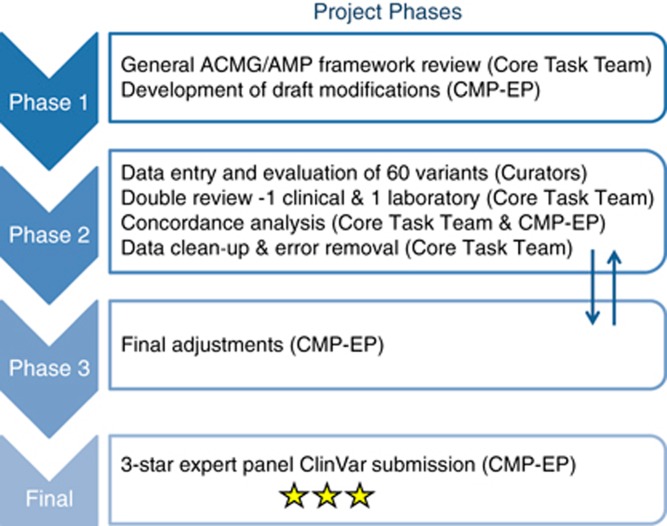
**Summary of ClinGen Inherited Cardiomyopathy Expert Panel (CMP-EP) involvement.** Phase 1: Disease/gene and other specifications made to established American College of Medical Genetics and Genomics/Association for Molecular Pathology (ACMG/AMP) framework. Phase 2: Selection and review of 60 pilot variants by two independent reviewers. Classifications were then compared and discussed to resolve any conflicts. Phase 3: Additional adjustments to variant classifications. Final: Expert panel variant classifications submitted to ClinVar for public accessibility. Expert panel ratings in ClinVar are denoted with a three-star rating.

An initial classification exercise included 10 variants and served as a foundation for subsequent rule adaptations. These variants were independently scored by members of the core task team using the original ACMG/AMP framework as well as their own institutions’ variant interpretation criteria. Subsequent work was carried out as shown in Figure 1. Draft rules were applied to 60 *MYH7* variants (including the initial 10), which were selected (i) as a representative spectrum of variant types for *MYH7*, (ii) to test as many rules as possible, (iii) to cover a range of classifications, and (iv) to include discrepant ClinVar assertions. Evidence for each variant was compiled by a set of curators (K.O., K.S., M.T.) and applicable rules were selected. Each variant was then reviewed by two task team members (one with clinical expertise and one with laboratory expertise; M.A.K., J.B., C.C., A.M.) and conflicts resulting from clerical errors and rule misuse were corrected. A discussion with the full CMP-EP was triggered when reviewers did not agree or raised concerns regarding the “fit” of a rule. Variants representing controversial items were used to drive additional rule adjustments by the CMP-EP. Adjusted rules were disseminated to the CMP-EP to allow for a final comment period. Additional guidance was provided by ClinGen’s Sequence Variant Interpretation Working Group, which harmonizes framework adaptation efforts by various clinical domain working groups (https://www.clinicalgenome.org/working-groups/genomic-variant-workgroup/sub-groups/sequence-variant-interpretation-wg/). Variants and the adapted rule framework were submitted to ClinVar under a 3-star (expert panel–reviewed) status.

### Curation data sources and data collection method (publicly available data)

Variants were curated using the variant assessment process and data sources described by Duzkale et al.^16^ (Supplementary Table S1). All databases accessed August to September 2015; reference transcript: NM_000257.3).

### Additional case-level data

The number of independent observations of the 60 pilot variants, basic phenotype information, and segregation with disease was available from several diagnostic or research cohorts (Partners HealthCare Laboratory for Molecular Medicine, Invitae, the Sarcomeric Human Cardiomyopathy Registry (https://theshareregistry.org/), the Australian Genetic Heart Disease Registry (http://www.heartregistry.org.au/), the National Institute for Health Research Cardiovascular Biomedical Research Unit at Royal Brompton Hospital and Imperial College London, and the National Heart Centre Singapore). Consideration was taken to account for cases that were known or suspected to be part of more than one cohort.

### Statistical approaches

#### Multiple proband rules (PS4, PS4_Moderate, PS4_Supporting)

The current ACMG/AMP framework assigns weight to increased prevalence of a variant in cases compared with controls (PS4), but does not provide guidance for combining separate studies reporting the same variant. We created thresholds of proband occurrences that qualify for supporting, moderate, and strong weight as follows and outlined in more detail in Supplementary Table S2. This approach represents a “quasi case-control” analysis. Proband cohort sizes were modeled based on the cohorts available for this study, focusing on Caucasian and African American ancestries (reflecting the main ancestries represented among cohorts available in this study). The corresponding ExAC cohorts were used as proxies for healthy controls. Odds ratios and *P* values were computed for 1–15 probands carrying a variant assuming absence/extreme rarity in controls (rule PM2 is met) using the two-sided Fisher’s exact test to evaluate the null hypothesis of conditional independence. This approach has limitations (for details see Supplementary Table 2) but provides a practical means for clinical variant assessment workflows.

#### Segregation thresholds

Since cardiomyopathies are characterized by variable age at onset and reduced penetrance, logarithm of odds (LOD) scores were estimated by counting the number of informative meioses separating affected variant carriers across all families with this variant (i.e., without considering unaffected individuals). Affected noncarriers indicated nonsegregation. Under these conditions the calculation of LOD score simplifies to LOD = log_10_(2^*n*^), where *n* is the number of informative meiosis observed.

## Results and discussion

*MYH7* is a major contributor to several cardiomyopathies (HCM, DCM, RCM). Due to their high combined prevalence and severe health outcomes, *MYH7* is one of the most frequently tested genes in a clinical setting. As a first step toward modifying the ACMG/AMP framework, the CMP-EP conducted a preliminary exercise comparing inter-expert concordance for 10 representative *MYH7* variants classified using the ACMG/AMP framework alongside the experts’ respective institutional criteria. As in similar previously reported studies,^7^ this revealed low inter-expert concordance (20%) for the ACMG/AMP framework compared with high concordance (90%) when institutional criteria were used (data not shown). The subsequent adaptation of the ACMG/AMP framework for *MYH7* was carried out as shown in Figure 1.

### Summary of specifications

Two characteristics of the ACMG/AMP framework had a major influence on the specifications made. Due to its design to have general applicability across all Mendelian disorders, some rules are overly conservative in the setting of a specific disorder. This is best illustrated by BA1, the allele frequency threshold above which a variant is considered benign. For many Mendelian conditions, the default threshold of 5% is orders of magnitude higher than it needs to be. In addition, the framework contains several areas of vagueness (such as the absence of quantitative guidance for increasing the weight depending on the extent of “segregation with disease”). Table 1 provides a summary of the adapted ACMG/AMP framework for use in *MYH7*-associated cardiomyopathies. Of the original 28 ACMG/AMP rules, 9 were deemed not applicable and another 12 required disease- and/or gene-specific adjustments. Five rules were given modified strength criteria. A full description of rules with additional detail is provided in Supplementary Text S2. The following sections highlight approaches and key specifications.

### Disease- and gene-specific adaptations

#### Minor allele frequency–driven rules (BA1, BS1, and PM2)

**Figure 2:**
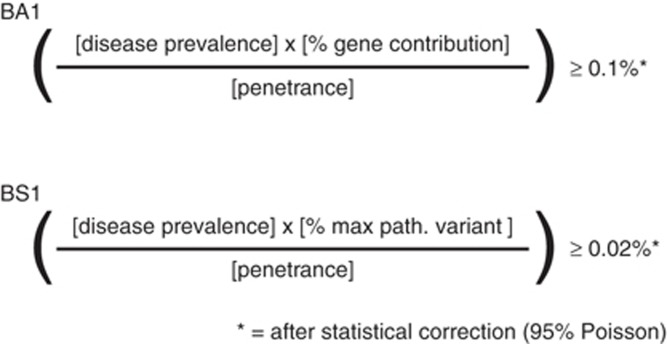
**Derivation of allele frequency thresholds for rules BA1 and BS1.** Disease prevalence = 1/200 individuals (1/400 chromosomes). Penetrance = 30%. % gene contribution = 10.6%. % maximum pathogenic variant contribution (max path. variant) = 2%.

The CMP-EP modified BA1 using extremely conservative values for disease prevalence, gene contribution, and estimated penetrance of *MYH7* variants (Figure 2, for additional detail see Supplementary Text S3). Across *MYH7*-associated diseases, prevalence values were compiled from the literature and the most conservative one was selected to derive a threshold that is applicable to all (1/200). Penetrance was set deliberately low at 30%. To control for uncertainty in estimated minor allele frequencies of very rare variants in smaller cohorts, a statistical correction (95% Poisson distribution) was added.^11^ This correction (termed “filtering allele frequency”) is now available for each variant for all ExAC cohorts, (http://exac.broadinstitute.org/). The final BA1 threshold was set at 0.1%. A variant that is observed at this minor allele frequency in the general population could theoretically be pathogenic under the assumption that it is the *only* pathogenic variant. Because of the extremely conservative approach, this threshold is likely two orders of magnitude higher than the true threshold and can be used safely in a diagnostic setting.

The threshold for BS1 (allele frequency too high for disorder) was derived using the same approach except that allelic heterogeneity was now considered (i.e., gene contribution was replaced by the maximum credible variant contribution, which encompasses both gene and variant contribution, Figure 2). This gives rise to a more aggressive threshold with less room for error, which is acceptable given the less definitive classification. The prevalence of the most common pathogenic cardiomyopathy variant was used to define the maximum theoretical population frequency for a pathogenic allele (Whiffin et al.^11^ and Supplementary Text S3). The final BS1 threshold was set at a filtering allele frequency of ≥0.02%. A variant observed at this minor allele frequency can be assumed to be likely benign provided that there is no substantial contradictory evidence supporting pathogenicity. Allowing a variant to reach a likely benign classification based on BS1 alone represents a revision of the original ACMG/AMP framework by ClinGen’s Sequence Variant Interpretation Working Group.^17^ The CMP-EP added the following safeguard to BS1: Because our current knowledge of the genetic architecture of HCM is largely derived from predominantly Caucasian proband cohorts, the threshold should only be applied to populations where sufficient numbers of probands have been deeply analyzed, leaving the possibility open that more common pathogenic variants may exist in less well-characterized populations. Both BA1 and BS1 were tested in a large diagnostic cohort (Partners HealthCare Laboratory for Molecular Medicine) and no currently known likely pathogenic or pathogenic *MYH7* variant would be misclassified as likely benign or benign (data not shown).

The CMP-EP recommends activating rule PM2 (absent in population databases) when the filtering allele frequency is <0.004%. The PM2 threshold used more realistic prevalence and penetrance values (1/500, 50%), which typically represents a very small/negligible number of alleles in the ExAC database.

#### Segregation with disease (PP1, PP1_Moderate, PP1_Strong)

The ACMG/AMP framework assigns supporting evidence to cosegregation (PP1) and states that higher weight can be assigned with an increasing degree of segregation, but does not define thresholds. The CMP-EP specified three levels of evidence using autosomal dominant likelihood ratios of 10 (3 meioses, LOD 0.9), 30 (5 meioses, LOD 1.5), and 100 (7 meioses, LOD 2.1) to count as supporting, moderate, and strong evidence provided that PM2 (absent or rare in large population cohorts) is met. Finally, the CMP-EP waived the ACMG/AMP recommendation for demonstrating segregation in more than one family given that *MYH7* is a well-established cardiomyopathy gene.

#### Increased prevalence of variant in probands versus controls (PS4, PS4_Moderate, PS4_Supporting)

Rule PS4 is designed for variants that are significantly enriched in probands. While traditional case-control studies using phenotyped case and control cohorts are typically not available for rare, Mendelian variants, it is not uncommon that multiple separate studies report the same variant in cohorts of modest size. To be able to utilize combined proband counts across different studies, the CMP-EP created a framework using the ExAC cohort as a proxy for healthy controls. As with segregation, evidence levels were assigned based on likelihood ratios with ideal target thresholds being 10 (supporting), 30 (moderate), and 100 (strong). To simplify use in current molecular diagnostic practice, where statistical tools are typically not embedded in routine workflows, conservative universal thresholds were set to proband counts of ≥2 (supporting), ≥6 (moderate), and ≥15 (strong). Moderate (but acceptable) deviations from the targeted odds ratios (ORs) for the two main racial cohorts used (NFE, non-Finnish European; AFR, African American) were deemed acceptable in return for ease of use (supporting: OR [AFR|NFE] = [10.4|13.4], moderate: OR [AFR|NFE] = [31.3|40.1], and strong: OR [AFR|NFE] = [79.1|100]). To apply these rules, rule PM2 must be met. These proband counts are extremely conservative to balance the limitations of the underlying statistical approach and the risk of double-counting probands that is inherent when working with published data.

#### De novo occurrence

Rule PS2 (de novo in a patient with the disease and no family history, paternity and maternity confirmed): The CMP-EP removed the requirement to prove maternity because the likelihood of undisclosed nonmaternity (e.g., due to surrogacy) was considered rare. In addition, the following specifications were added: (i) “no family history” is defined as the absence of diagnosed disease or suspicious findings in a three-generation pedigree and (ii) both parents must be genotype and phenotype negative after a thorough clinical evaluation that ideally includes a combination of electrocardiogram and echo or cardiac magnetic resonance imaging for maximum sensitivity (Supplementary Text S2). When paternity has not been established, de novo occurrence receives moderate weight (PM6) but the CMP-EP allowed upgrading to “strong” (PS2) when at least three de novo occurrences have been documented.

#### Dealing with ambiguous phenotypes when counting multiple or segregation with disease

**Left ventricular noncompaction:** It is currently debated whether left ventricular noncompaction is a distinct cardiomyopathy, a morphological trait shared by different cardiomyopathies, or an entirely benign structural variant.^18, 19, 20^ The CMP-EP therefore recommended that individuals with isolated left ventricular noncompaction (no additional cardiomyopathy such as HCM or DCM present) should *not* be added to proband or segregation counts in the context of HCM and DCM. **End-stage HCM**: Due to the challenge in distinguishing between end-stage HCM and DCM, a conservative approach was taken to not include DCM cases in proband or segregation counts for HCM variants, unless earlier clinical evidence supported the HCM phenotype.

#### Functional data

The ACMG/AMP framework assigns strong weight to well-established *in vitro* or *in vivo* functional studies that are supportive of a damaging effect on the gene or protein (PS3). The normal function of the protein encoded by *MYH7* is to convert energy from adenosine triphosphate hydrolysis into mechanical force to allow for muscle contractility.^21^ After reviewing the assays used to measure this function for the 60 pilot variants, the CMP-EP determined that “strong functional evidence” can only be provided by a mammalian variant-specific knock-in model (Supplementary Table S3). Other *in vivo* models that alter dosage of the normal protein (transgenic or knockout mice, zebrafish knock-downs) are not acceptable as they do not provide clues about the importance of a particular variant. Typically performed *MYH7* *in vitro* assays were generally deemed to have relatively low positive predictive value (either due to low accuracy or low correlation between an observed effect and the ability to cause disease) and are therefore currently not considered strong evidence. However, the CMP-EP recognized that in the event that *in vitro* models that accurately predict the effect *in vivo* become available, their weight can be reconsidered.

#### Incorporating protein domain information

The ACMG/AMP framework assigns supporting evidence of pathogenicity to missense variants in a gene that has a low rate of missense variation, provided that missense variants are a common mechanism of disease (PP2) and moderate evidence for variants located in a hotspot and/or critical domain without benign variation (PM1). It is well established that missense variants in *MYH7* are the predominant class of pathogenic alleles. The ExAC database (http://exac.broadinstitute.org/^22^) provides a metric to express the deviation of variant counts from the expected number (constraint score). Positive scores indicate intolerance to variation, which is the case for *MYH7* (*z* = 6.54). However, recent studies suggest that this is driven by statistically significant clustering of pathogenic variants in the head region (*P* < 3  ×  10^−15^, amino acids 181–937, NM_000257).^23, 24^ The CMP-EP concluded that this evidence was most appropriately weighted as moderate through application of the critical domain rule (PM1). PP2 was deemed no longer applicable because it does not apply to variants outside the head domain and to avoid double counting the same evidence twice for variants in the head domain.

### Other modifications

#### Rules deemed not applicable

Four rules of the pathogenic framework (PVS1, PM3, PP2, PP4) and three rules of the benign framework (BS2, BP1, BP3) were deemed not applicable either entirely or in the original strength level suggested, and two additional rules were removed for other reasons. Select rules are discussed here and a full list of not applicable and removed rules, along with a summary of the rationale, is provided in Supplementary Text S2. PVS1 (null variant in a gene where loss of function (LOF) is a known disease mechanism): *MYH7* LOF variants are very rare and their contribution to inherited cardiomyopathy is incompletely understood. While there is currently no evidence for a disease-causing role in the heterozygous state, compound heterozygosity of LOF variants along with missense variants can lead to extremely severe presentations, mimicking recessive inheritance.^25, 26^ The CMP-EP assigned moderate weight to a LOF variant (PVS1_Moderate, Table 1), which yields a classification of variant of uncertain significance in the absence of case-level data supporting pathogenicity. PM3 (variant detected in *trans* with a pathogenic variant): While compound heterozygosity leading to a more severe phenotype has been documented, this rule was designed for traditional recessive inheritance.

#### Removed rules

PP5/BP6 (reputable source reports variant as pathogenic/benign, but evidence is not accessible): The CMP-EP decided expert curations should only be used if accompanied by the evidence used. Platforms such as ClinVar enable laboratories to share the evidence on which an interpretation is based, and the CMP-EP encourages this practice.

### Performance of the new *MYH7*-specific rules

Sixty pilot variants were selected to cover a broad spectrum of scenarios, while focusing on the types of data most commonly encountered (Supplementary Figure S1, Supplementary Table S4). The majority of the rules were applied at least once (Supplementary Figure S2). The rules supporting benign evidence were used the least, reflecting the intentional bias toward the pathogenic spectrum. After application of the modified ACMG/AMP framework by two independent expert reviewers, 8/60 variants were discordantly classified. Factors underlying discordance revealed two causes: (i) 4 data errors (differences in the data used, such as arriving at different proband or segregation counts), and (ii) 4 rule applications that deviated from the intended use (Supplementary Figure S3). After correcting for data errors, concordance was 93%. Rules requiring a high level of expert knowledge were more vulnerable to error (e.g., those relying on segregation counts), exposing a significant overhead associated with training curators. All discrepancies were resolved upon review by the task team and did not require full CMP-EP review. This represents a significant improvement compared with the initial 10 variant pilot. The pilot variants (31 associated with HCM and 6 with DCM) have been submitted to ClinVar with a 3-star (expert panel–approved) label. ClinVar IDs are listed in Supplementary Table S4.

### Laboratory internal data has a high impact and shows a critical need to enable sharing of case-level information

**Figure 3:**
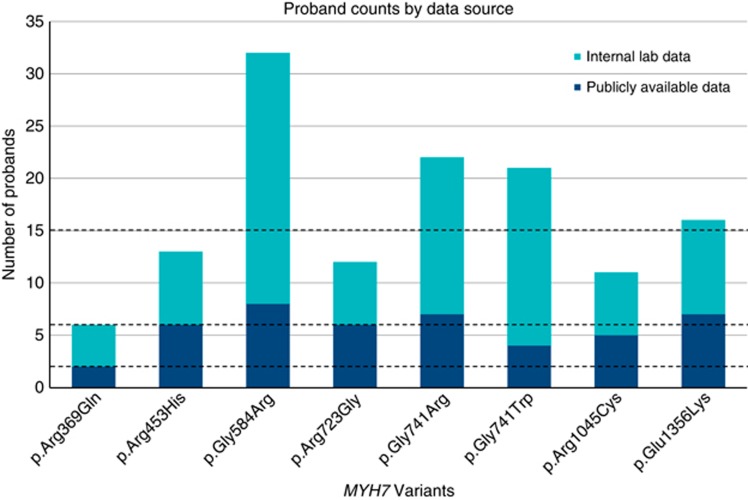
**Impact of data sharing on proband counts.** Increased proband counts obtained from internal lab data changed the variant classification for eight variants. Hashed lines correspond to the thresholds for supporting (≥2, PS4_Supporting), moderate (≥6, PS4_Moderate), and strong (≥15, PS4). Publicly available data was collected from PubMed, Google, Human Gene Mutation Database Professional, ClinVar, and relevant locus-specific variant databases. Internal laboratory data was collected from the Partners HealthCare Laboratory for Molecular Medicine, Invitae, the Sarcomeric Human Cardiomyopathy Registry (https://theshareregistry.org/), the Australian Genetic Heart Disease Registry (http://www.heartregistry.org.au/), the National Institute for Health Research Cardiovascular Biomedical Research Unit at Royal Brompton Hospital and Imperial College London, and the National Heart Centre Singapore.

When available, the CMP-EP also reviewed internal laboratory data from several of its members that was unpublished at the time of curation (see Materials and Methods). For 25 pilot variants, this impacted the application or strength for at least one of the multiple proband, segregation, or de novo rules and impacted the final classification of 12 variants (48%). Seven variants were upgraded from likely pathogenic to pathogenic, and another five increased from uncertain significance to likely pathogenic (Figure 3, Supplementary Table S5). This is a powerful demonstration of the impact of historically decentralized, private data and illustrates an urgent need to incentivize data sharing as well as to establish infrastructure and standards to share and aggregate such case-level data.

### Clinical judgment

Although the ACMG/AMP framework represents a major step forward in our ability to classify variants in the context of Mendelian disease, it will need continued improvement and refinement as our understanding of these diseases develops. It is premature to expect that even this improved, rule-based framework will function without additional clinical judgment, which is a hallmark of medical practice. Clinical judgment represents the capability of experts to consider additional, as of yet unquantified factors, and adjust the weighting of specific evidence elements intuitively based on deep experience and exposure to many cases over time. Prior studies have begun to quantitate this phenomenon in the context of variant classification.^7^ In our pilot, judgment led to overriding the classification for two *MYH7* variants where the CMP-EP upgraded the rule-based classification of variant of uncertain significance to likely pathogenic: p.Arg1420Trp (PM2, absent/rare in controls; PS4_Moderate, present in 11 probands; PP3, computational predictions favor pathogenic) and p.Arg1909Pro (PM2, absent/rare in controls; PM6, de novo occurrence without confirmed paternity; PP3, computational predictions favor pathogenic). Considering the extremely conservative approaches used for several rules, the CMP-EP felt that the evidence for both variants was sufficiently borderline (just one additional supporting rule required to meet the criteria for likely pathogenic) and the available evidence provided additional specificity not accounted for by the rules to warrant this upgrade (p.Arg1420Trp, additional probands not counted due to conservative nature of the approach; p.Arg1909Pro, phenotype included DCM and myopathy, and additional segregations).

## Limitations

Proband cohorts used to derive data on multiple occurrences of variants largely represent diagnostic cases from broad referral populations where clinical diagnoses were based on information provided by ordering health-care providers.

Allele frequency thresholds were developed assuming autosomal dominant inheritance and were deliberately designed as overly stringent to minimize the risk for false-positive interpretations, which can cause harm to the patients and their families.

In accordance with the ACMG/AMP parent framework, these rules assume a single-variant disease paradigm.

## Conclusions and future directions

The CMP-EP created adjusted ACMG/AMP variant classification rules that can be used for all *MYH7*-associated cardiomyopathies. It is expected that our framework will undergo iterative refinements catalyzed by its use, as well as continuous improvements made to the parent framework by the community, and the CMP-EP will provide updated versions as needed. Future work will extend the *MYH7* framework to other cardiomyopathy genes, which will likely require only minimal additional specifications.

Finally, the CMP-EP will establish a sustained curation process to apply the *MYH7* rules to all variants presently in the public domain with the goal to submit 3-star (expert panel–reviewed) variant classifications along with the associated evidence into ClinVar.

## Figures and Tables

**Table 1:** Summary of the adapted ACMG/AMP pathogenic and benign criteria

**Pathogenic criteria**			
**Rule**		**Specification type**	**Rule description**
VS	PVS1	Removed	Null variant in gene with established LOF as disease mechanism
Strong	PS1	No change	Different nucleotide change (same amino acid) as a previously established pathogenic variant
	PS2	Disease/gene	De novo (paternity confirmed) in a patient with disease and no family history
	PS3	Disease/gene	Functional studies of mammalian knock-in models supportive of a damaging effect on the gene or gene product
	PS4	Disease/gene	Prevalence of the variant in affected individuals is significantly increased compared with the prevalence in controls - OR - Variant identified in ≥15 probands with consistent phenotypes
	PP1_Strong	Modif. strength	Variant segregates with ≥7 meioses
Moderate	PM1	Disease/gene	Hotspot/est. functional domain (amino acids 181–937) without benign variation
	PM2	Disease/gene	Absent/extremely rare (<0.004%) from large population studies
	PM3	Removed	Detected in *trans* with a pathogenic variant (recessive)
	PM4	No change	Protein length changes due to in-frame deletions/insertions of any size in a nonrepeat region or stop-loss variants
	PM5	No change	Missense change at an amino acid residue where a different missense change previously established as pathogenic
	PM6	Disease/gene	Confirmed de novo without confirmation of paternity
	PVS1_Moderate	Modif. strength	Null variant in gene with evidence supporting LOF as disease mechanism
	PS4_Moderate	Modif. strength	Variant identified in ≥6 probands with consistent phenotypes
	PP1_Moderate	Modif. strength	Variant segregates in ≥5 meioses
Supporting	PP1	Disease/gene	Variant segregates in ≥3 meioses
	PP2	Removed	Missense variant in a gene that has a low rate of benign missense variation and where missense variants are a common mechanism of disease
	PP3	No change	Multiple lines of computational evidence support a deleterious effect on the gene or gene product
	PP4	Removed	Phenotype specific for disease with single genetic etiology
	PP5	Removed	Reputable source reports as pathogenic
	PS4_Supporting	Modif. strength	Variant identified in ≥2 probands with consistent phenotypes
**Benign criteria**			
**Rule**		**Specification type**	**Rule description**
SA	BA1	Disease/gene	Allele frequency is ≥0.1% based on the filtering allele frequency in ExAC
Strong	BS1	Disease/gene	Allele frequency is ≥0.02% based on the filtering allele frequency in ExAC provided there is no conflicting information
	BS2	Removed	Observed in healthy adult with full penetrance expected at an early age
	BS3	No change	Functional studies of mammalian knock-in models supportive of no damaging effect on protein function or splicing
	BS4	Disease/gene	Nonsegregation in affected members of a family
Supporting	BP1	Removed	Missense variant in gene where only LOF causes disease
	BP2	Disease/gene	Observed as comp het (in *trans*) or double het in genes with overlapping function (e.g., sarcomere genes) without increased disease severity *or* observed in *cis* with a pathogenic variant in any inheritance pattern
	BP3	Removed	In-frame deletions/insertions in a repetitive region without a known function
	BP4	No change	Multiple lines of computational evidence suggest no impact on gene or gene product
	BP5	Disease/gene	Variant found in a case with an alternate molecular basis for disease
	BP6	Removed	Reputable source reports as benign
	BP7	No change	A silent variant for which splicing prediction algorithms predict no impact to the splice consensus sequence nor the creation of a new splice site -AND- the nucleotide is not highly conserved

ACMG/AMP, American College of Medical Genetics and Genomics/Association for Molecular Pathology; LOF, loss of function; Modif. strength, modified rule strength; Removed, not applicable to *MYH7*-associated disease; SA, standalone; VS, very strong.

Numbers under each classification refer to the number of rules with that strength required to classify the variant as its header category. Example: a likely pathogenic classification may be made with one piece of strong and two pieces of supporting evidence.
