# Supplementary material for: Adaptation and validation of the ACMG/AMP variant classification framework for MYH7-associated inherited cardiomyopathies: recommendations by ClinGen’s Inherited Cardiomyopathy Expert Panel
Source: Genet Med. 2018 Jan 4;20(3):351–9. doi: 10.1038/gim.2017.218 (PMC5876064; doi:10.1038/gim.2017.218)
Supplement: Supplementary Figures [file gim2017218x1.pdf]

SUPPLEMENTARY FIGURES

A. Variants by classification

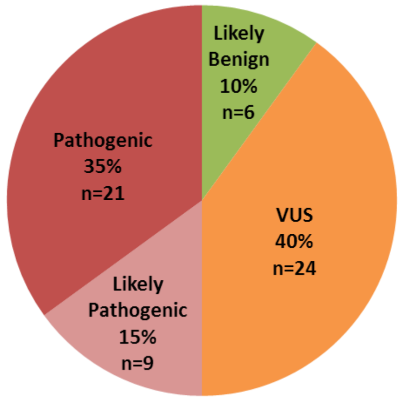

B. Summary of ClinVar classification discrepancies

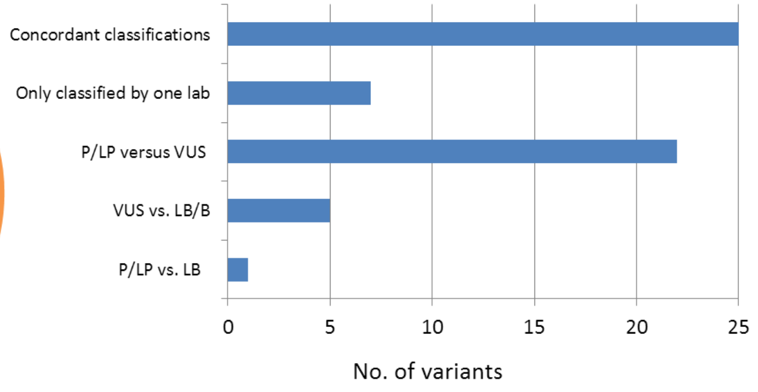

C. Variant change type

**Variant Type**	**N**
Missense	50
FS	1
Silent	2
Intronic	2
Deletion	3
Splice	1
Nonsense	1
**Total**	60

Supplementary Figure 1. Summary statistics for 60 *MYH7* pilot variants at the time of variant selection. Variants with a broad spectrum of classifications and evidence levels were selected. **A.** Classifications of 60 pilot variants from the Partners Laboratory for Molecular Medicine (LMM) at the time of variant selection. **B.** Discrepancies in classifications listed in ClinVar between main cardiomyopathy labs. P = Pathogenic, LP = Likely Pathogenic, VUS = Variant of uncertain significance, LB = Likely benign, B = Benign. **C.** Breakdown of 60 pilot variants by type.

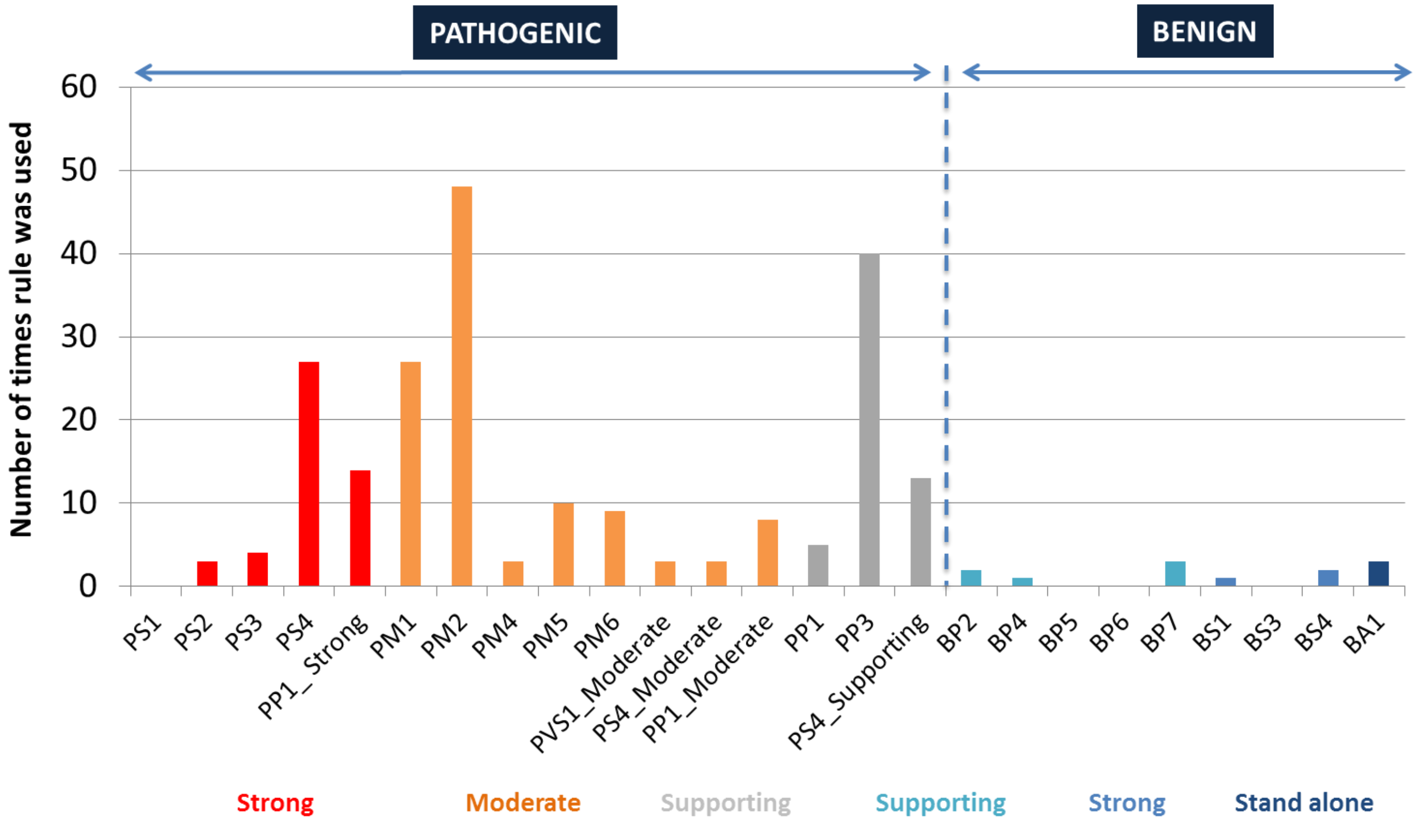

Supplementary Figure 2. Summary of rules that were applied to the 60 *MYH7* variants. The not applicable or removed rules are not included above. The variants covered a range of classifications from pathogenic to benign and allowed for 21/25 rules to be tested.

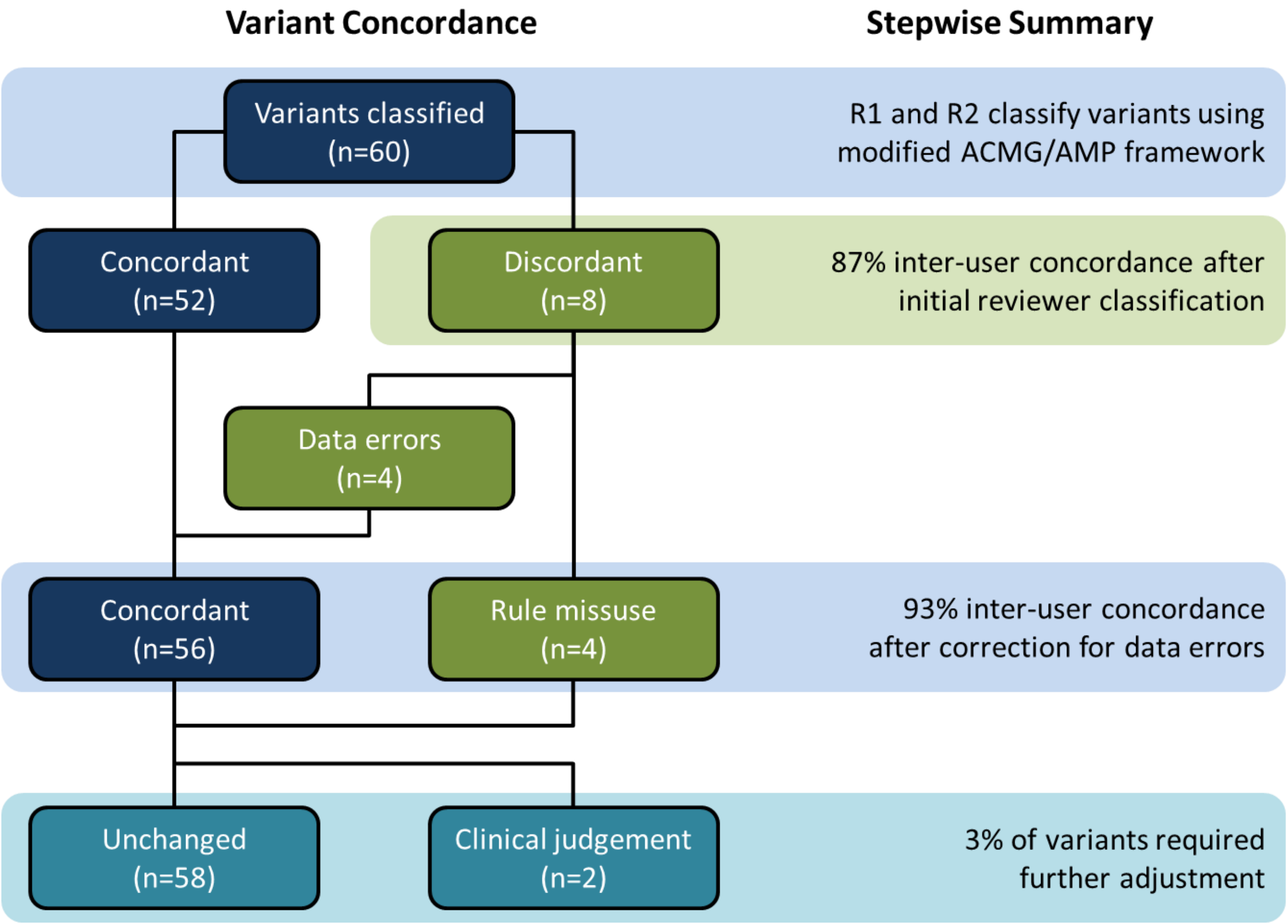

Supplementary Figure 3: Summary of underlying factors for variant classification discordance. Numbers in (n) represent the number of variants. R1/R2 = Reviewers 1 and 2.
