# Supplementary material for: Adaptation and validation of the ACMG/AMP variant classification framework for MYH7-associated inherited cardiomyopathies: recommendations by ClinGen’s Inherited Cardiomyopathy Expert Panel
Source: Genet Med. 2018 Jan 4;20(3):351–9. doi: 10.1038/gim.2017.218 (PMC5876064; doi:10.1038/gim.2017.218)
Supplement: Supplementary Tables [file gim2017218x2.pdf]

SUPPLEMENTARY TABLES

Supplementary Table 1. Data sources and elements used for variant classification.

Alamut® was used to derive the following variant annotations:

Annotation	Cell Name	Value
Gene symbol	Alamut.gene	*MYH7*
Gene id (HGNC)	Alamut.geneId	*7577*
Chromosome	Alamut.chrom	*14*
Transcript	Alamut.transcript	*NM_000257.2*
Strand	Alamut.strand	*-1*
Transcript Length	Alamut.transLen	-
Protein	Alamut.protein	*NP_000248.2*
Uniprot accession	Alamut.uniprot	-
Variant Type	Alamut.varType	*substitution*
Variant coding effect	Alamut.codingEffect	*missense*
Variant location	Alamut.varLocation	*exon*
Genome assembly	Alamut.assembly	*GRCh37*
gDNA start	Alamut.gDNAstart	*23884860*
gDNA end	Alamut.gDNAend	*23884860*
Genomic-level nomenclature	Alamut.gNomen	*g.23884860C>T*
cDNA start	Alamut.cDNAstart	*5135*
cDNA end	Alamut.cDNAend	*5135*
CDNA-level nomenclature	Alamut.cNomen	*c.5135G>A*
Protein-level nomenclature	Alamut.pNomen	*p.Arg1712Gln*

Additional annotations and tools used:

- MAF (ExAC cohort) – http://exac.broadinstitute.org/
- Computational Prediction Tools: Align GVGD[1], PolyPhen2 (HumVar)[2], MAPP[3], SIFT[4], Mutation Taster[5], Sarcomere Polyphen[6]
- Splice Prediction Tools: SpliceSiteFinder-like[7], MaxEntScan[8], Splice Site Prediction by Neural Network (NNSPLICE)[9], GeneSplicer[10], Human Splicing Finder[11]
- Published case data was compiled from PubMed, Google, HGMD, ClinVar, and relevant locus specific variant databases (Leiden Muscular Dystrophy: http://www.dmd.nl/ and LOVD: http://www.genomed.org/lovd2/home.php)
- Available case-level data from participating laboratories/research efforts included proband counts for variants examined as well as basic clinical information (clinical diagnosis as specified by the ordering provider, segregation with disease)

Supplementary Table 2. Derivation of proband count thresholds.

We created thresholds of proband occurrences that qualify for supporting, moderate, and strong weight as follows.

Aggregate numbers of probands were compared to ExAC cohorts, which serve as proxies for healthy controls. Briefly, odds ratios and p-values were computed using the two-sided Fisher's exact test to evaluate the null hypothesis of conditional independence and was done for Caucasian and African American probands. This was done for Caucasian (n=5,000) and African American (n=1,000) probands, reflecting the main ancestries represented among cohorts available in this study. The corresponding Exome Aggregation Consortium (ExAC) populations were African/African American (AFR, n=5,203) and Non-Finnish European (NFE, n=33,370). Test statistics as well as p-values were computed separately.

This approach has limitations including the assumption that ExAC is a suitable control population and does not account for heterogeneity across cohorts/separate studies, which was balanced by choosing stringent parameters and the general nature of the framework, which generally requires evidence from several types of data to elevate a variant to a clinically significant status (pathogenic or likely pathogenic classification).

Proband Cohort Size		1000						5000					
Analysis		p-values			odds ratios (cases:ctrls)			p-values			odds ratios (cases:ctrls)		
Exac Population		African (n=5203)			African (n=5203)			Non-Finnish Euro (n=33370)			Non-Finnish Euro (n=33370)		
Alleles in Eur. ExAC		0	1	2	0	1	2	0	1	2	0	1	2
Proband Counts													
Supporting	2	0.02596761	0.06954428	0.12438762	Infinity	10.4	5.2	0.01697778	0.04651012	0.08503795	Infinity	13.4	6.7
	3	0.00417927	0.01470093	0.0323598	Infinity	15.6	7.8	0.00221161	0.00798229	0.01802046	Infinity	20.0	10.0
	4	0.00067205	0.00292835	0.00766265	Infinity	20.9	10.4	0.00028804	0.00129019	0.00346929	Infinity	26.7	13.4
Moderate	5	0.00010798	0.0005612	0.00170257	Infinity	26.1	13.1	3.75E-05	0.00020064	0.0006263	Infinity	33.4	16.7
	6	1.73E-05	0.00010466	0.00036126	Infinity	31.3	15.7	4.88E-06	3.04E-05	0.00010796	Infinity	40.1	20.0
	7	2.78E-06	1.91E-05	7.40E-05	Infinity	36.7	18.3	6.36E-07	4.51E-06	1.80E-05	Infinity	46.8	23.4
	8	4.46E-07	3.44E-06	1.48E-05	Infinity	41.9	21.0	8.27E-08	6.59E-07	2.91E-06	Infinity	53.5	26.7
	9	7.14E-08	6.11E-07	2.88E-06	Infinity	47.2	23.6	1.08E-08	9.51E-08	4.62E-07	Infinity	60.2	30.1
Strong	10	1.14E-08	1.07E-07	5.51E-07	Infinity	52.5	26.2	1.40E-09	1.36E-08	7.19E-08	Infinity	66.9	33.4
	11	1.83E-09	1.87E-08	1.04E-07	Infinity	57.8	28.9	1.82E-10	1.93E-09	1.10E-08	Infinity	73.6	36.8
	12	2.91E-10	3.23E-09	1.93E-08	Infinity	63.1	31.5	2.37E-11	2.71E-10	1.67E-09	Infinity	80.3	40.1
	13	4.65E-11	5.55E-10	3.55E-09	Infinity	68.5	34.3	3.08E-12	3.79E-11	2.50E-10	Infinity	87.0	43.4
	14	7.42E-12	9.47E-11	6.45E-10	Infinity	73.8	36.9	4.01E-13	5.28E-12	3.71E-11	Infinity	93.7	46.9
	15	1.18E-12	1.61E-11	1.16E-10	Infinity	79.1	39.6	5.21E-14	7.32E-13	5.46E-12	Infinity	100.4	50.2

p-values Key
>0.05
0.001 to 0.05
0.0001 to 0.001
<0.0001

Supplementary Table 3: Summary of functional assays for 60 *MYH7* pilot variants.

For 23 of the 60 variants functional assays were described. Types of assays are specified for each *MYH7* variant. PS3 was applied only to variants observed to have variant specific knock-in mouse models (p.Arg453Cys, p.Arg403Gln, p.Arg719Trp, p.Ser532Pro).

	**Analysis Method**	***MYH7* Variant**	**References**
**In vitro**	Motility assays	p.Arg403Gln, p.Arg403Trp, p.Arg453Cys, p.Arg719Gln, p.Arg719Trp, p.Arg870His, p.Asp906Gly, p.Gly584Arg, p.Gly716Arg, p.Leu908Val, p.Ser532Pro	12-25
	Protein quantification (Western blot, etc)	p.Arg403Gln, p.Arg403Trp, p.Arg663His, p.Arg719Gln, p.Asn1327Lys, p.Glu1356Lys, p.Glu1768Lys, p.Gly741Arg, p.Ile736Thr, p.Leu908Val, p.Lys847Glu	12,19,24,26-28
	Optical trap	p.Arg403Gln, p.Arg403Trp, p.Arg453Cys, p.Arg719Gln, p.Arg719Trp, p.Gly584Arg, p.Gly716Arg, p.Leu908Val, p.Ser532Pro	13,16,20,22,25
	ATPase assays	p.Arg403Gln, p.Arg403Trp, p.Arg453Cys, p.Arg719Gln, p.Arg719Trp, p.Gly584Arg, p.Gly716Arg	13,16,20-25,29
	mRNA quantification (qPCR, Northern blot, etc)	p.Arg403Gln, p.Arg719Trp, p.Arg723Gly, p.Asp1096Tyr, p.Ile736Thr, p.Thr1377Met	24,30-36
	Functional measurements (Skinned fibers, cardiomyocytes, or myofibrils)	p.Arg403Gln, p.Arg453Cys, p.Arg719Trp, p.Arg723Gly, p.Ile736Thr, p.Ser532Pro	21,33,34,37-41
	Liquid chromatography/ Mass spectrometry	p.Arg719Trp, p.Arg723Gly, p.Asp1096Tyr, p.Ile736Thr, p.Thr1377Met	32,35,42
	Spectroscopy (circular dichroism, NMR, etc)	p.Arg403Gln, p.Arg870His, p.Asn1327Lys, p.Glu1356Lys, p.Glu1768Lys	26,43-45
	Cell attachment/contractility	p.Arg403Gln, p.Asn1327Lys, p.Glu1356Lys, p.Glu1768Lys	26,46
	Fluorescence recovery after photobleaching	p.Asn1327Lys, p.Glu1356Lys, p.Glu1768Lys	26
	Co-sedimentation experiments	p.Arg870His, p.Ser1776Gly	43,47
	Stopped flow system	p.Arg403Gln, p.Arg453Cys	48,49
	Isovolumic contractile performance	p.Arg403Gln, p.Ser532Pro	45,50,51
	Thermodynamic analysis (thermal denaturation, isothermal titration calorimetry)	p.Glu1356Lys, p.Arg870His	43,44
	Ca2+ imaging and whole-cell patch clamping	p.Arg663His	52
	Light scattering	p.Glu1356Lys	44
**In vivo**	Variant specific knock-in mouse model	p.Arg453Cys, p.Arg403Gln, p.Arg719Trp, p.Ser532Pro	22,36,53-55
	Transgenic animal model (cDNA based)	p.Arg403Gln	56
	Magnetic resonance spectroscopy	p.Arg403Gln	57-59
	Isovolumic contractile performance	p.Arg403Gln	50

Supplementary Table 4: Summary of pilot variant analyses

This table is provided as a separate supplemental document

Supplementary Table 5: Impact of laboratory internal data on proband counts and segregation data

Variant			Proband Counts			Segregation Counts			De Novo Counts			Impact on Classification
Genomic	cDNA	Amino Acid	Public Sources	Internal Data	Total	Public Sources	Internal Data	Total	Public Sources	Internal Data	Total	
g.23898213C>T	c.1358G>A	p.Arg453His	6	7	13	-	-	-	1	-	1	
g.23896932C>G	c.1750G>C	p.Gly584Arg	8	24	32	3	2	5	-	-	-	
g.23895023G>A	c.2167C>T	p.Arg723Cys	9	11	20	4	3	7	1	-	1	
g.23894983A>G	c.2207T>C	p.Ile736Thr	16	10	26	4	1	5	-	-	-	LP --> P
g.23894969C>G	c.2221G>C	p.Gly741Arg	7	15	22	3	1	4	1	-	-	
g.23894969C>A	c.2221G>T	p.Gly741Trp	4	17	21	4	2	6	-	-	-	
g.23893357T>C	c.2681A>G	p.Glu894Gly	16	13	29	-	6	6	-	-	-	
g.23899016C>T	c.1106G>A	p.Arg369Gln	2	4	6	-	2	2	-	1	1	
g.23898538T>C	c.1157A>G	p.Tyr386Cys	1	-	1	-	-	-	-	2	2	
g.23891501G>A	c.3133C>T	p.Arg1045Cys	5	6	11	-	1	1	-	-	-	VUS --> LP
g.23887522C>T	c.4066G>A	p.Glu1356Lys	7	9	16	-	5	5	-	-	-	
g.23884362C>T	c.5401G>A	p.Glu1801Lys	1	3	4	2	-	2	-	2	2	

Publical data sources included PubMed, Google, HGMD Professional, ClinVar, and relevant locus specific variant databases. Internal laboratory data was available from the Partners HealthCare Laboratory for Molecular Medicine, Invitae, Inc, the Sarcomeric Human Cardiomyopathy Registry - SHaRe, https://theshareregistry.org/, the Australian Genetic Heart Disease Registry - http://www.heartregistry.org.au/, the NIHR Cardiovascular Biomedical Research Unit at Royal Brompton Hospital and Imperial College London, and the National Heart Centre Singapore). P refers to a pathogenic classification; LP refers to a likely pathogenic classification; VUS refers to a classification of uncertain significance.

References

1. Mathe E, Olivier M, Kato S, Ishioka C, Hainaut P, Tavtigian SV. Computational approaches for predicting the biological effect of p53 missense mutations: a comparison of three sequence analysis based methods. *Nucleic Acids Res.* 2006;34(5):1317-1325.
2. Adzhubei IA, Schmidt S, Peshkin L, et al. A method and server for predicting damaging missense mutations. *Nat Methods.* 2010;7(4):248-249.
3. Stone EA, Sidow A. Physicochemical constraint violation by missense substitutions mediates impairment of protein function and disease severity. *Genome Res.* 2005;15(7):978-986.
4. Ng PC, Henikoff S. Predicting deleterious amino acid substitutions. *Genome Res.* 2001;11(5):863-874.
5. Schwarz JM, Cooper DN, Schuelke M, Seelow D. MutationTaster2: mutation prediction for the deep-sequencing age. *Nat Methods.* 2014;11(4):361-362.
6. Jordan DM, Kiezun A, Baxter SM, et al. Development and validation of a computational method for assessment of missense variants in hypertrophic cardiomyopathy. *Am J Hum Genet.* 2011;88(2):183-192.
7. Shapiro MB, Senapathy P. RNA splice junctions of different classes of eukaryotes: sequence statistics and functional implications in gene expression. *Nucleic Acids Res.* 1987;15(17):7155-7174.
8. Yeo G, Burge CB. Maximum entropy modeling of short sequence motifs with applications to RNA splicing signals. *J Comput Biol.* 2004;11(2-3):377-394.

9. Reese MG, Eeckman FH, Kulp D, Haussler D. Improved splice site detection in Genie. *J Comput Biol.* 1997;4(3):311-323.
10. Pertea M, Lin X, Salzberg SL. GeneSplicer: a new computational method for splice site prediction. *Nucleic Acids Res.* 2001;29(5):1185-1190.
11. Desmet FO, Hamroun D, Lalande M, Collod-Beroud G, Claustres M, Beroud C. Human Splicing Finder: an online bioinformatics tool to predict splicing signals. *Nucleic Acids Res.* 2009;37(9):e67.
12. Keller DI, Coirault C, Rau T, et al. Human homozygous R403W mutant cardiac myosin presents disproportionate enhancement of mechanical and enzymatic properties. *J Mol Cell Cardiol.* 2004;36(3):355-362.
13. Yamashita H, Tyska MJ, Warshaw DM, Lowey S, Trybus KM. Functional consequences of mutations in the smooth muscle myosin heavy chain at sites implicated in familial hypertrophic cardiomyopathy. *J Biol Chem.* 2000;275(36):28045-28052.
14. Wang Q, Moncman CL, Winkelmann DA. Mutations in the motor domain modulate myosin activity and myofibril organization. *J Cell Sci.* 2003;116(Pt 20):4227-4238.
15. Cuda G, Fananapazir L, Epstein ND, Sellers JR. The in vitro motility activity of beta-cardiac myosin depends on the nature of the beta-myosin heavy chain gene mutation in hypertrophic cardiomyopathy. *J Muscle Res Cell Motil.* 1997;18(3):275-283.
16. Fujita H, Sugiura S, Momomura S, Omata M, Sugi H, Sutoh K. Characterization of mutant myosins of Dictyostelium discoideum equivalent to human familial hypertrophic cardiomyopathy mutants. Molecular force level of mutant myosins may have a prognostic implication. *J Clin Invest.* 1997;99(5):1010-1015.
17. Jacques AM, Briceno N, Messer AE, et al. The molecular phenotype of human cardiac myosin associated with hypertrophic obstructive cardiomyopathy. *Cardiovasc Res.* 2008;79(3):481-491.
18. Alpert NR, Mohiddin SA, Tripodi D, et al. Molecular and phenotypic effects of heterozygous, homozygous, and compound heterozygote myosin heavy-chain mutations. *Am J Physiol Heart Circ Physiol.* 2005;288(3):H1097-1102.
19. Cuda G, Fananapazir L, Zhu WS, Sellers JR, Epstein ND. Skeletal muscle expression and abnormal function of beta-myosin in hypertrophic cardiomyopathy. *J Clin Invest.* 1993;91(6):2861-2865.
20. Palmiter KA, Tyska MJ, Haeberle JR, Alpert NR, Fananapazir L, Warshaw DM. R403Q and L908V mutant beta-cardiac myosin from patients with familial hypertrophic cardiomyopathy exhibit enhanced mechanical performance at the single molecule level. *J Muscle Res Cell Motil.* 2000;21(7):609-620.
21. Palmer BM, Fishbaugher DE, Schmitt JP, et al. Differential cross-bridge kinetics of FHC myosin mutations R403Q and R453C in heterozygous mouse myocardium. *Am J Physiol Heart Circ Physiol.* 2004;287(1):H91-99.
22. Debold EP, Schmitt JP, Patlak JB, et al. Hypertrophic and dilated cardiomyopathy mutations differentially affect the molecular force generation of mouse alpha-cardiac myosin in the laser trap assay. *Am J Physiol Heart Circ Physiol.* 2007;293(1):H284-291.
23. Tyska MJ, Hayes E, Giewat M, Seidman CE, Seidman JG, Warshaw DM. Single-molecule mechanics of R403Q cardiac myosin isolated from the mouse model of familial hypertrophic cardiomyopathy. *Circ Res.* 2000;86(7):737-744.
24. Di Domenico M, Casadonte R, Ricci P, et al. Cardiac and skeletal muscle expression of mutant beta-myosin heavy chains, degree of functional impairment and phenotypic heterogeneity in hypertrophic cardiomyopathy. *J Cell Physiol.* 2012;227(10):3471-3476.
25. Sommese RF, Sung J, Nag S, et al. Molecular consequences of the R453C hypertrophic cardiomyopathy mutation on human beta-cardiac myosin motor function. *Proc Natl Acad Sci U S A.* 2013;110(31):12607-12612.
26. Wolny M, Colegrave M, Colman L, White E, Knight PJ, Peckham M. Cardiomyopathy mutations in the tail of beta-cardiac myosin modify the coiled-coil structure and affect integration into thick filaments in muscle sarcomeres in adult cardiomyocytes. *J Biol Chem.* 2013;288(44):31952-31962.
27. Theis JL, Bos JM, Theis JD, et al. Expression patterns of cardiac myofilament proteins: genomic and protein analysis of surgical myectomy tissue from patients with obstructive hypertrophic cardiomyopathy. *Circ Heart Fail.* 2009;2(4):325-333.
28. Leung MC, Hitchen PG, Ward DG, Messer AE, Marston SB. Z-band alternatively spliced PDZ motif protein (ZASP) is the major O-linked beta-N-acetylglucosamine-substituted protein in human heart myofibrils. *J Biol Chem.* 2013;288(7):4891-4898.
29. Roopnarine O, Leinwand LA. Functional analysis of myosin mutations that cause familial hypertrophic cardiomyopathy. *Biophys J.* 1998;75(6):3023-3030.

30. Borchert B, Tripathi S, Francino A, Navarro-Lopez F, Kraft T. The left and right ventricle of a patient with a R723G mutation of the beta-myosin heavy chain and severe hypertrophic cardiomyopathy show no differences in the expression of myosin mRNA. *Cardiol J.* 2010;17(5):518-522.
31. Witjas-Paalberends ER, Ferrara C, Scellini B, et al. Faster cross-bridge detachment and increased tension cost in human hypertrophic cardiomyopathy with the R403Q MYH7 mutation. *J Physiol.* 2014;592(15):3257-3272.
32. Helms AS, Davis FM, Coleman D, et al. Sarcomere mutation-specific expression patterns in human hypertrophic cardiomyopathy. *Circ Cardiovasc Genet.* 2014;7(4):434-443.
33. Kraft T, Witjas-Paalberends ER, Boontje NM, et al. Familial hypertrophic cardiomyopathy: functional effects of myosin mutation R723G in cardiomyocytes. *J Mol Cell Cardiol.* 2013;57:13-22.
34. McKee LA, Chen H, Regan JA, et al. Sexually dimorphic myofilament function and cardiac troponin I phosphospecies distribution in hypertrophic cardiomyopathy mice. *Arch Biochem Biophys.* 2013;535(1):39-48.
35. Tripathi S, Schultz I, Becker E, et al. Unequal allelic expression of wild-type and mutated beta-myosin in familial hypertrophic cardiomyopathy. *Basic Res Cardiol.* 2011;106(6):1041-1055.
36. Teekakirikul P, Eminaga S, Toka O, et al. Cardiac fibrosis in mice with hypertrophic cardiomyopathy is mediated by non-myocyte proliferation and requires Tgf-beta. *J Clin Invest.* 2010;120(10):3520-3529.
37. Kirschner SE, Becker E, Antognozzi M, et al. Hypertrophic cardiomyopathy-related beta-myosin mutations cause highly variable calcium sensitivity with functional imbalances among individual muscle cells. *Am J Physiol Heart Circ Physiol.* 2005;288(3):H1242-1251.
38. Seebohm B, Matinmehr F, Kohler J, et al. Cardiomyopathy mutations reveal variable region of myosin converter as major element of cross-bridge compliance. *Biophys J.* 2009;97(3):806-824.
39. Blanchard E, Seidman C, Seidman JG, LeWinter M, Maughan D. Altered crossbridge kinetics in the alphaMHC403/+ mouse model of familial hypertrophic cardiomyopathy. *Circ Res.* 1999;84(4):475-483.
40. Kohler J, Winkler G, Schulte I, et al. Mutation of the myosin converter domain alters cross-bridge elasticity. *Proc Natl Acad Sci U S A.* 2002;99(6):3557-3562.
41. Belus A, Piroddi N, Scellini B, et al. The familial hypertrophic cardiomyopathy-associated myosin mutation R403Q accelerates tension generation and relaxation of human cardiac myofibrils. *J Physiol.* 2008;586(15):3639-3644.
42. Becker E, Navarro-Lopez F, Francino A, Brenner B, Kraft T. Quantification of mutant versus wild-type myosin in human muscle biopsies using nano-LC/ESI-MS. *Anal Chem.* 2007;79(24):9531-9538.
43. Gruen M, Gautel M. Mutations in beta-myosin S2 that cause familial hypertrophic cardiomyopathy (FHC) abolish the interaction with the regulatory domain of myosin-binding protein-C. *J Mol Biol.* 1999;286(3):933-949.
44. Armel TZ, Leinwand LA. A mutation in the beta-myosin rod associated with hypertrophic cardiomyopathy has an unexpected molecular phenotype. *Biochem Biophys Res Commun.* 2010;391(1):352-356.
45. Spindler M, Saupe KW, Christe ME, et al. Diastolic dysfunction and altered energetics in the alphaMHC403/+ mouse model of familial hypertrophic cardiomyopathy. *J Clin Invest.* 1998;101(8):1775-1783.
46. Chuan P, Sivaramakrishnan S, Ashley EA, Spudich JA. Cell-intrinsic functional effects of the alpha-cardiac myosin Arg-403-Gln mutation in familial hypertrophic cardiomyopathy. *Biophys J.* 2012;102(12):2782-2790.
47. Flashman E, Watkins H, Redwood C. Localization of the binding site of the C-terminal domain of cardiac myosin-binding protein-C on the myosin rod. *Biochem J.* 2007;401(1):97-102.
48. Bloemink M, Deacon J, Langer S, et al. The hypertrophic cardiomyopathy myosin mutation R453C alters ATP binding and hydrolysis of human cardiac beta-myosin. *J Biol Chem.* 2014;289(8):5158-5167.
49. Lowey S, Bretton V, Gulick J, Robbins J, Trybus KM. Transgenic mouse alpha- and beta-cardiac myosins containing the R403Q mutation show isoform-dependent transient kinetic differences. *J Biol Chem.* 2013;288(21):14780-14787.
50. Georgakopoulos D, Christe ME, Giewat M, Seidman CM, Seidman JG, Kass DA. The pathogenesis of familial hypertrophic cardiomyopathy: early and evolving effects from an alpha-cardiac myosin heavy chain missense mutation. *Nat Med.* 1999;5(3):327-330.
51. Palmer BM, Schmitt JP, Seidman CE, et al. Elevated rates of force development and MgATP binding in F764L and S532P myosin mutations causing dilated cardiomyopathy. *J Mol Cell Cardiol.* 2013;57:23-31.
52. Lan F, Lee AS, Liang P, et al. Abnormal calcium handling properties underlie familial hypertrophic cardiomyopathy pathology in patient-specific induced pluripotent stem cells. *Cell Stem Cell.* 2013;12(1):101-113.
53. Blankenburg R, Hackert K, Wurster S, et al. beta-Myosin heavy chain variant Val606Met causes very mild hypertrophic cardiomyopathy in mice, but exacerbates HCM phenotypes in mice carrying other HCM mutations. *Circ Res.* 2014;115(2):227-237.

54. Geisterfer-Lowrance AA, Christe M, Conner DA, et al. A mouse model of familial hypertrophic cardiomyopathy. *Science.* 1996;272(5262):731-734.
55. Schmitt JP, Debold EP, Ahmad F, et al. Cardiac myosin missense mutations cause dilated cardiomyopathy in mouse models and depress molecular motor function. *Proc Natl Acad Sci U S A.* 2006;103(39):14525-14530.
56. Abraham MR, Bottomley PA, Dimaano VL, et al. Creatine kinase adenosine triphosphate and phosphocreatine energy supply in a single kindred of patients with hypertrophic cardiomyopathy. *Am J Cardiol.* 2013;112(6):861-866.
57. Vikstrom KL, Factor SM, Leinwand LA. Mice expressing mutant myosin heavy chains are a model for familial hypertrophic cardiomyopathy. *Mol Med.* 1996;2(5):556-567.
58. Lowey S, Lesko LM, Rovner AS, et al. Functional effects of the hypertrophic cardiomyopathy R403Q mutation are different in an alpha- or beta-myosin heavy chain backbone. *J Biol Chem.* 2008;283(29):20579-20589.
59. Cammarato A, Li XE, Reedy MC, Lee CF, Lehman W, Bernstein SI. Structural basis for myopathic defects engendered by alterations in the myosin rod. *J Mol Biol.* 2011;414(4):477-484.
