# Supplementary material for: Adaptation and validation of the ACMG/AMP variant classification framework for MYH7-associated inherited cardiomyopathies: recommendations by ClinGen’s Inherited Cardiomyopathy Expert Panel
Source: Genet Med. 2018 Jan 4;20(3):351–9. doi: 10.1038/gim.2017.218 (PMC5876064; doi:10.1038/gim.2017.218)
Supplement: Supplementary Text 1 [file gim2017218x3.pdf]

SUPPLEMENTARY TEXT 1

Decision Process for Specifications to the ACMG/AMP Framework

1) Initial proposed adjustments developed via conference calls and emails.
2) Electronic survey (see below) was used to gather feedback on all proposed changes from members of the Cardiomyopathy Expert Panel and larger Cardiovascular Domain Working Group.
    a. Members could agree, disagree, propose additional adjustments and leave other comments.
3) Cardiomyopathy Expert Panel members votes were tallied and majority was required (≥2/3 majority) for approval.
    a. All rules that did not meet consensus requirements were discussed via conference calls and emails, and additional adjustments were made.
    b. All Cardiomyopathy Expert Panel members reviewed and approved the final adjustments.

Example of Electronic Survey Questions

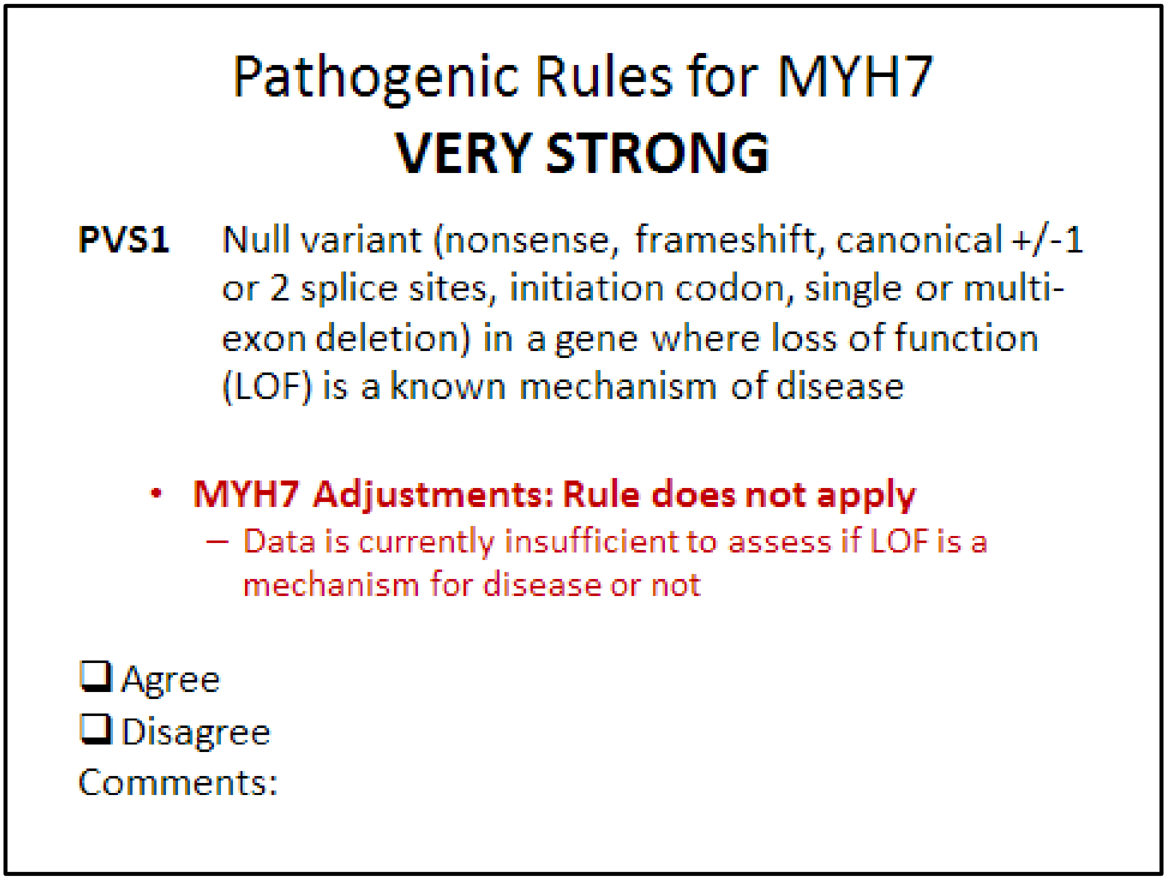

Pathogenic Rules for MYH7
STRONG

PS1 Same amino acid change as a previously established pathogenic variant regardless of nucleotide change

- *Example:* Val->Leu caused by either G>C or G>T in the same codon
- *Caveat:* Beware of changes that impact splicing rather than at the amino acid/protein level

- **MYH7 Adjustments:** None, apply as stated

❑ Agree
❑ Disagree
Comments:

Pathogenic Rules for MYH7
STRONG

PS2 De novo (both maternity and paternity confirmed) in ***a patient with the disease*** and ***no family history***

- *Note:* Confirmation of paternity only is insufficient. Egg donation, surrogate motherhood, errors in embryo transfer, etc. can contribute to non-maternity.
- All cases assumed de novo, but without confirmation of paternity and maternity will be a **moderate** criteria **(PM6)**

- **MYH7 Adjustments:**
  - Only paternity required (likelihood of non-disclosed non-maternity is very low)
  - No family history requires a minimum of parents to have been screened (ECHOs) and a "non-suspicious" family history (e.g. no SCD, hx of transplant, ICD, features of CM)

❑ Agree
❑ Disagree
Comments:
