# Supplementary material for: Adaptation and validation of the ACMG/AMP variant classification framework for MYH7-associated inherited cardiomyopathies: recommendations by ClinGen’s Inherited Cardiomyopathy Expert Panel
Source: Genet Med. 2018 Jan 4;20(3):351–9. doi: 10.1038/gim.2017.218 (PMC5876064; doi:10.1038/gim.2017.218)
Supplement: Supplementary Text 2 [file gim2017218x4.pdf]

ClinGen Inherited Cardiomyopathy Expert Panel (CMP-EP)
Modified ACMG/AMP Classification Rules for *MYH7*

SUMMARY OF CLASSIFICATION CRITERIA

Pathogenic Criteria				Combination of Criteria Needed to Meet Classification							
	Rule	Specification Type	Rule Description	Pathogenic				Likely Pathogenic			
VS	PVS1	Removed	~~Null variant in gene with established LOF as disease mechanism~~								
STRONG	PS1	No change	Different nucleotide change (same amino acid) as a previously established pathogenic variant	2	1	1	1	1	1		
	PS2	Disease/gene	*De novo (* paternity confirmed) in a patient with disease and no family history								
	PS3	Disease/gene	Functional studies of mammalian knock-in models supportive of a damaging effect on the gene or gene product								
	PS4	Disease/gene	Prevalence of the variant in affected individuals is significantly increased compared to the prevalence in controls -OR- Variant identified in ≥15 probands with consistent phenotypes								
	PP1_Strong	Modif. Strength	Variant segregates with ≥7 meioses								
MODERATE	PM1	Disease/gene	Hotspot/est. functional domain (amino acids 181-937) without benign variation		3	2	1	1		3	2
	PM2	Disease/gene	Absent/extremely rare (<0.004%) from large population studies								
	PM3	Removed	~~Detected in trans with a pathogenic variant (recessive)~~								
	PM4	No change	Protein length changes due to in-frame deletions/insertions of any size in a non-repeat region or stop-loss variants								
	PM5	No change	Missense change at an amino acid residue where a different missense change previously established as pathogenic								
	PM6	Disease/gene	Confirmed *de novo* without confirmation of paternity								
	PVS1_Moderate	Modif. Strength	Null variant in gene with evidence supporting LOF as disease mechanism								
	PS4_Moderate	Modif. Strength	Variant identified in ≥6 probands with consistent phenotypes								
	PP1_Moderate	Modif. Strength	Variant segregates in ≥5 meioses								
SUPPORTING	PP1	Disease/gene	Variant segregates in ≥3 meioses			2	4		2		2
	PP2	Removed	~~Missense variant in a gene that has a low rate of benign missense variation and where missense variants are a common mechanism of disease~~								
	PP3	No change	Multiple lines of computational evidence support a deleterious effect on the gene or gene product								
	PP4	Removed	~~Phenotype specific for disease with single genetic etiology~~								
	PP5	Removed	~~Reputable source reports as pathogenic~~								
	PS4_Supporting	Modif. strength	Variant identified in ≥2 probands with consistent phenotypes								

Benign Criteria				Combination of Criteria Needed to Meet Classification			
	Rule	Specification Type	Rule Description	Benign		Likely Benign	
SA	BA1	Disease/gene	Allele frequency is ≥0.1% based on the filtering allele frequency (FAF) in ExAC	1			
STRONG	BS1	Disease/gene	Allele frequency is ≥0.02% based on the filtering allele frequency (FAF) in ExAC provided there is no conflicting information		2	1	
	BS2	Removed	~~Observed in healthy adult with full penetrance expected at an early age~~				
	BS3	No change	Functional studies of mammalian knock-in models supportive of no damaging effect on protein function or splicing				
	BS4	Disease/gene	Non-segregation in affected members of a family				
SUPPORTING	BP1	Removed	~~Missense variant in gene where only LOF causes disease~~				2
	BP2	Disease/gene	Observed as comp het (in trans) or double het in genes with overlapping function (e.g. sarcomere genes) without increased disease severity -OR- Observed in cis with a pathogenic variant in any inheritance pattern				
	BP3	Removed	~~In-frame deletions/insertions in a repetitive region without a known function~~				
	BP4	No change	Multiple lines of computational evidence suggest no impact on gene or gene product				
	BP5	Disease/gene	Variant found in a case with an alternate molecular basis for disease				
	BP6	Removed	~~Reputable source reports as benign~~				
	BP7	No change	A silent variant for which splicing prediction algorithms predict no impact to the splice consensus sequence nor the creation of a new splice site -AND- the nucleotide is not highly conserved				

VS = Very Strong. SA = Stand alone. Removed - not applicable to *MYH7* -associated disease. Modif. strength: Modified rule strength. Numbers under each classification refer to the number of rules with that strength required to classify the variant as its header category. Example: A likely pathogenic classification may be made with 1 piece of strong and 2 pieces of supporting evidence.

RULES FOR COMBINING CRITERIA

PATHOGENIC

1. 1 Very Strong AND
    a. ≥1 Strong OR
    b. ≥2 Moderate OR
    c. 1 Moderate and 1 Supporting OR
    d. ≥2 Supporting
2. ≥2 Strong OR
3. 1 Strong AND
    a. ≥3 Moderate OR
    b. 2 Moderate AND ≥2 Supporting OR
    c. 1 Moderate AND ≥4 Supporting

LIKELY PATHOGENIC

1. 1 Very Strong AND 1 Moderate OR
2. 1 Strong AND 1-2 Moderate OR
3. 1 Strong AND ≥2 Supporting OR
4. ≥3 Moderate OR
5. 2 Moderate AND ≥2 Supporting OR
6. 1 Moderate AND ≥4 Supporting

BENIGN

1. 1 Stand-Alone OR
2. ≥2 Strong

LIKELY BENIGN

1. 1 Strong* OR
2. ≥2 Supporting

** Allowing a variant to reach a likely benign classification based on BS1 alone represents a revision of the original ACMG/AMP framework by ClinGen's Sequence Variant Interpretation (SVI) Working Group.*

DETAILED DESCRIPTIONS OF CLASSIFICATION CRITERIA - PATHOGENICITY

Note: The rules from the original ACMG/AMP framework that were deemed not applicable or removed are listed separately at the end of this document, including a summary for why each rule was not applicable or removed.

STRONG EVIDENCE OF PATHOGENICITY

PS1 **Same amino acid change as a previously established pathogenic variant (as defined by these modified guidelines) regardless of nucleotide change.**

Example: Val->Leu caused by either G>C or G>T in the same codon.
Caveat: Beware of changes that impact splicing rather than at the amino acid/protein level.
Note: *MYH7* splice variants are exceedingly rare.

PS2 ***De novo*** **(paternity confirmed) in <u>a patient with the disease</u> and <u>no family history.</u>**

- Both parents must have been tested for the variant and shown to not carry the variant.
- Paternally inherited rare variants are sufficient to evaluate the possibility of non-paternity (e.g. A proband has several rare VUSs identified and the father also carries some of the rare VUSs).
- Generally only applicable in the <u>ABSENCE</u> of any other possible disease-causing variants. If other suspicious variants are present, recommend using as *MODERATE* criterion (see PM6 below).
- No family history requires that parents have been thoroughly clinically evaluated without evidence suggestive of cardiomyopathy (ideally using a combination of ECG and echocardiogram or cardiac MRI for maximum sensitivity).
    a. Three-generation family history should not include any cardiomyopathy reported in a 1st or 2nd degree relative.
    b. Examples of family history concerning for/suggestive of cardiomyopathy include any of the following in a 1st or 2nd degree relative:
        1. Sudden death under 60 years of age
        2. Heart transplant
        3. Implantable cardiac defibrillator (ICD) under 60 years of age
        4. Features of cardiomyopathy (e.g. systolic dysfunction, hypertrophy, left ventricular enlargement in an individual without risk factors). One should have confidence in the accuracy of clinical features.
        5. Other related/overlapping cardiomyopathies
        6. Gene-specific phenotypes should be kept in mind (e.g. conduction system disease or myopathy with *LMNA*)
    c. Examples of non-suspicious family history may include non-specific clinical features (e.g. palpitations, syncope, borderline/inconclusive echocardiogram findings, heart attack if age appropriate and suspected to result from coronary artery disease), but every attempt should be made to clarify features.

PS3 **Well-established *in vitro* or *in vivo* functional studies supportive of a damaging effect on the gene or gene product.**

Note: Functional studies that have been validated and shown to be reproducible and robust in a clinical diagnostic laboratory setting are considered to be well-established.

- Functional data sufficient to be considered as strong evidence:
    a. Currently, only mammalian variant-specific knock-in models are considered sufficient for *MYH7*, while other *in vivo* evidence (such as whole gene alterations) are not acceptable.
    b. A cell model (or other *in vitro* assay) may provide strong evidence where
        i. the variant in question produces a cellular phenotype that reliably predicts the clinical phenotype, and
        ii. causality is demonstrated with appropriate controls (e.g. correction of the variant reverses the phenotype). Not yet available for *MYH7*-related cardiomyopathy, but may be applicable in the future.
- Strength of all functional studies should be assessed for a high-specificity for the assay/model as well as for the associated phenotype being sufficiently rare and the direction of effect on the gene product consistent with what is known for that disease (i.e. GOF vs LOF).

PS4 **<u>Scenario 1:</u> The prevalence of the variant in affected individuals is significantly increased compared to the prevalence in controls.**

Note: Apply ONLY when a well-designed case-control study has been performed).

- Relative risk (RR) or odds ratio (OR), as obtained from case-control studies, is >5.0 and the confidence interval around the estimate of RR or OR does not include 1.0. See manuscript for detailed guidance.
- Controls should have been thoroughly clinically evaluated without evidence suggestive of cardiomyopathy (ideally using a combination of ECG and echocardiogram or cardiac MRI for maximum sensitivity).
- Any variant with published statistical calculations (p-value, OR, RR) or variant where this rule could possibly be applied should be reviewed for statistical validity.
- It is recommended that this rule **NOT** be used for variants that have been reported in multiple independent patient cohorts. See **scenario 2** below, and rules **PS4_Moderate and PS4_Supporting** on how to assign weight to multiple proband occurrences.

Scenario 2: Variant identified in multiple probands with consistent phenotypes (between probands and for the gene): *STRONG* evidence requires ≥15 probands with consistent phenotypes; can lower this to 10 if it has been verified that all probands are unrelated.

- Only applicable if variant is absent or rare in large population studies (PM2 criteria met).
- Phenotypes should be clinically confirmed and should not include individuals with a suspected diagnosis.
- Proband counts may combine cases of HCM and RCM. The combination of probands with other phenotypes should be reviewed by a clinical expert to determine if their inclusion in proband counts is appropriate.
- LVNC and end stage HCM:
  a. Generally, individuals with isolated LVNC should NOT be added to proband or segregation counts (including individuals with isolated LVNC in a family where other cardiomyopathy is present).
  b. Due to the challenge in distinguishing between end-stage HCM and DCM, caution should be exercised when considering DCM cases for inclusion in proband or segregation counts for primarily HCM variants.
- Threshold of ≥15 probands was deliberately chosen to be stringent to counteract the risk for double counting probands (the inclusion of the same individuals in >1 published study is not always disclosed) and an option for reducing the strong threshold to ≥10 was factored in if such double counting can be *definitively* ruled out.

PP1_Strong Co-segregation with disease in multiple affected family members in a gene definitively known to cause the disease: *STRONG* evidence requires ≥7 segregations (LOD score of ~2.1, or ~1/100 occur by chance).

- Only applicable if variant is also absent or rare in large population studies (PM2 criteria met).
- Only genotype positive/phenotype positive individuals are counted as segregations. Genotype positive/phenotype negative individuals are generally less informative for *MYH7*-associated disorders due to variable age at onset and reduced penetrance.
- *CAUTION* needed when counting segregations in presence of other possible disease-causing variants.
- *CAUTION* needed when family members are distantly related (≥3rd degree) and affected individuals are linked by unknown or unaffected relatives (raises possibility of multiple causes of disease).

MODERATE EVIDENCE OF PATHOGENICITY

PM1 Located in a mutational hot spot and/or critical and well-established functional domain (e.g. active site of an enzyme) without benign variation (as defined by these modified guidelines)

- Applicable when missense variant is located within the head domain (codons 181-937, NM_000257).

PM2 Absent from controls or at extremely low frequency in Exome Sequencing Project, 1000 Genomes or ExAC.

- A filtering allele frequency (FAF) <0.004% activates this rule
- *Caveat*: Due to current technical limitations, minor allele frequencies for complex variants (e.g. large indels) may not be accurately represented in population databases that are based on next generation sequencing technologies.
  - Position must have sufficient coverage (≥20x) in each control individual being counted.
  - Race/ethnicity of reported cases should be included in the populations covered by Exome Sequencing Project, 1000 Genomes, ExAC, or gnomAD. Caution should be used when a variant is absent from population data and has been only seen in individuals of an uncommon minority, as that ethnicity may not be well represented in the population databases and be falsely absent.
  - *CAUTION*: Population databases may contain presymptomatic individuals for diseases with reduced penetrance/variable onset.

PM4 Protein length changes due to in-frame deletions/insertions of any size in a non-repeat region or stop-loss variants.

PM5 Missense change at an amino acid residue where a different missense change determined to be pathogenic (as defined by these modified guidelines) has been seen before.

Example: p.Arg156His is pathogenic; now you observe p.Arg156Cys.

Caveat: Beware of changes that impact splicing rather than at the amino acid/protein level. However, *MYH7* splice variants are exceedingly rare.

PM6 Confirmed *de novo*, but without confirmation of paternity and maternity.

- Family history must be consistent with *de novo* occurrence, though clinical evaluation of parents is not required.
- Both parents must have been tested and shown to not carry the variant.
- If other suspicious variants are present, recommend only using as MODERATE criterion, even if paternity is confirmed.
- Do not use if PS2 has been applied.
- If a variant satisfies criteria for **PM6** AND has been observed to occur *de novo* ≥3 times, then *de novo* evidence may be elevated to *STRONG (**PS2**)*.

PVS1_Moderate Null variant (nonsense, frameshift, canonical +/-1 or 2 splice sites, initiation codon, single or multi-exon deletion) in a gene where there is evidence supporting, but it has not been established that loss of function (LOF) as a mechanism of disease.

Caveats:

- Use caution interpreting LOF variants at the extreme 3' end of a gene
- Use caution with splice variants that are predicted to lead to exon skipping, but leave the remainder of the protein intact
- Use caution in the presence of multiple transcripts

PS4_Moderate Variant identified in multiple probands with consistent phenotypes (between probands and for the gene): *MODERATE* evidence requires ≥6 probands with consistent phenotypes.

- Do not use when PS4 (strong) has been applied.
- Only applicable if variant is absent or rare in large population studies (PM2 criteria met).
- Phenotypes should be clinically confirmed and should not include individuals with a suspected diagnosis.
- Proband counts may combine cases of HCM and RCM. The combination of probands with other phenotypes should be reviewed by a clinical expert to determine if their inclusion in proband counts is appropriate.
- LVNC and end stage HCM:
  a. Generally, individuals with isolated LVNC should NOT be added to proband or segregation counts (including individuals with isolated LVNC in a family where other cardiomyopathy is present).
  b. Due to the challenge in distinguishing between end-stage HCM and DCM, caution should be exercised when considering DCM cases for inclusion in proband or segregation counts for primarily HCM variants.

PP1_Moderate Co-segregation with disease in multiple affected family members in a gene definitively known to cause the disease: *MODERATE* evidence requires ≥5 segregations (LOD score of ~1.5, or ~1/30 occur by chance)

- Only applicable if variant is also absent or rare in large population studies (PM2 criteria met).
- Only genotype positive/phenotype positive individuals are counted as segregations. Genotype positive/phenotype negative individuals are generally less informative for *MYH7*-associated disorders due to variable age at onset and reduced penetrance).
- *CAUTION* needed when counting segregations in presence of other possible disease-causing variants.
- *CAUTION* needed when distantly related (≥3rd degree) affected individuals are connected by unknown or unaffected relatives (raises possibility of multiple causes of disease).

SUPPORTING EVIDENCE OF PATHOGENICITY

PP1 Co-segregation with disease in multiple affected family members in a gene definitively known to cause the disease: *SUPPORTING* evidence requires ≥3 segregations (LOD score of ~0.9, or ~1/10 occur by chance).

- Only applicable if variant is also absent or rare in large population studies (PM2 criteria met).
- Only genotype positive/phenotype positive individuals are counted as segregations. Genotype positive/phenotype negative individuals are generally less informative for *MYH7*-associated disorders due to variable age at onset and reduced penetrance).
- *CAUTION* needed when counting segregations in presence of other possible disease-causing variants.

- *CAUTION* needed when distantly related (≥3rd degree) affected individuals are connected by unknown or unaffected relatives (raises possibility of multiple causes of disease).

PP3 Multiple lines of computational evidence support a deleterious effect on the gene or gene product (conservation, evolutionary, splicing impact, etc).

Caveat: As many in silico algorithms use the same or very similar input for their predictions, each algorithm should not be counted as an independent criterion. PP3 can be used only once in any evaluation of a variant.
Note: Positive predictive value for benign/no impact predictions is higher than for pathogenic/impact predictions.

PS4_Supporting Variant identified in multiple probands with consistent phenotypes (between probands and for the gene): *SUPPORTING* evidence requires ≥2 probands with consistent phenotype.

- Do not use when PS4 (strong) or PS4_Moderate has been applied.
- Only applicable if variant is absent or rare in large population studies (PM2 criteria met).
- Phenotypes should be clinically confirmed and should not include individuals with a suspected diagnosis.
- Proband counts may combine cases of HCM and RCM. The combination of probands with other phenotypes should be reviewed by a clinical expert to determine if their inclusion in proband counts is appropriate.
- Phenotypes should be clinically confirmed and should not include individuals with a suspected diagnosis.
- LVNC and end stage HCM:
  a. Generally, individuals with isolated LVNC should NOT be added to proband or segregation counts (including individuals with isolated LVNC in a family where other cardiomyopathy is present).
  b. Due to the challenge in distinguishing between end-stage HCM and DCM, caution should be exercised when considering DCM cases for inclusion in proband or segregation counts for primarily HCM variants.

DETAILED DESCRIPTIONS OF CLASSIFICATION CRITERIA – BENIGN IMPACT

STAND-ALONE EVIDENCE OF BENIGN IMPACT

BA1 Allele frequency is ≥0.1% based on the filtering allele frequency (FAF) listed in ExAC.

STRONG EVIDENCE OF BENIGN IMPACT

BS1 Allele frequency is ≥0.02% based on the filtering allele frequency (FAF) in ExAC, which is greater than expected for *MYH7*-associated cardiomyopathies.

CAUTION: This threshold should only be applied to populations where a sufficient number of probands has been deeply analyzed.

BS3 Well-established *in vitro* or *in vivo* functional studies shows no damaging effect on protein function or splicing.

Note: Functional studies that have been validated and shown to be reproducible and robust in a clinical diagnostic laboratory setting are considered the most well-established

- Functional data sufficient to be considered strong evidence:
  a. Currently mammalian knock-in models are considered sufficient for *MYH7*, while other in vivo evidence (such as whole gene alterations) are not acceptable.
  b. A cell model (or other *in vitro* assay) may provide strong evidence where
     i. the variant in question produces a cellular phenotype that reliably predicts the clinical phenotype, and
     ii. causality is demonstrated with appropriate controls (e.g. correction of the variant reverses the phenotype). Not yet available for *MYH7*-related cardiomyopathy, but may be applicable in the future.
- Strength of all functional studies should be assessed for a high-specificity for the assay/model as well as for the phenotype with the variant being sufficiently rare and the direction of effect on the gene product consistent with what is known for that disease (i.e. GOF vs LOF).

BS4 Lack of segregation (non-segregation) in affected members of a family.

Caveat: The presence of phenocopies for common phenotypes (i.e. cancer, epilepsy) can mimic lack of segregation among affected individuals. Also, families may have more than one pathogenic variant contributing to an autosomal dominant disorder, further confounding an apparent lack of segregation.

- Requires careful consideration of when to use, due to possible phenocopies and families with multiple pathogenic variants.
    a. Any non-segregations should be carefully evaluated to rule out a phenocopy or the presence of a second disease-causing variant before considering it as conflicting or benign evidence.
- Generally useful for downgrading a VUS to likely benign/benign. Careful consideration is required when using this data as conflicting evidence, especially when overall evidence supports likely pathogenic or pathogenic.
- Due to the possibility of phenocopies and the possibility of multiple disease-causing variants, minimum of 2 non-segregations without a possible alternate cause required to apply criteria.

SUPPORTING EVIDENCE OF BENIGN IMPACT

BP2 Observed as compound heterozygous (in trans) or double heterozygous in genes with overlapping function (e.g. sarcomere genes) without increased disease severity or observed in cis with a pathogenic variant in any inheritance pattern.

- Other variants should be pathogenic as defined by these modified guidelines.
- Testing of parents or other informative relatives is required to determine cis/trans status.
- If a variant is seen in trans or double heterozygous with another pathogenic variant in two or more cases (2+) and the phenotype is not more severe than when either of the two variants are seen in isolation, this rule may be applied.
    a. <1% of cases of HCM have >1 pathogenic or likely pathogenic variant (0.6%, Alfares 2015, PubMed ID 25611685).
- This rule cannot be applied when the variant has only ever been observed in cis with the pathogenic variant as its significance in isolation is unknown in this scenario.
- *CAUTION* should be used when this rule is the primary piece of evidence for classifying a variant as likely benign/benign (only 2 supporting criteria are sufficient for a likely benign classification).

BP4 Multiple lines of computational evidence suggest no impact on gene or gene product (conservation, evolutionary, splicing impact, etc).

Caveat: As many in silico algorithms use the same or very similar input for their predictions, each algorithm cannot be counted as an independent criterion. BP4 can be used only once in any evaluation of a variant.

BP5 Variant found in a case with an alternate molecular basis for disease.

- Due to high variability in penetrance and expressivity for *MYH7*-associated cardiomyopathies as well as the non-negligible prevalence of individuals with more than one pathogenic variant, application of this rule requires additional consideration such as the severity/onset of the disorder relative to what would be expected on average.
    a. Likely only applicable in rare cases.
    b. Likely only when proband's phenotype is not within the spectrum expected for this gene and other alternate cause clearly explains phenotype.
    c. Do NOT apply if variant may at all be contributing to disease (e.g. increased severity or phenotype not fully explained by alternate cause).

BP7 A synonymous (silent) variant OR intronic variant outside the splice consensus sequence (-4 and +7 outward) for which splicing prediction algorithms predict no impact to the splice consensus sequence NOR the creation of a new splice site AND the nucleotide is not highly conserved.

Note: Rule can be combined with BP4 to make a variant LB.

NOT APPLICABLE OR REMOVED RULES

PVS1 (null variant in a gene where loss of function (LOF) is a known disease mechanism): *MYH7* LOF variants are very rare and their contribution to inherited cardiomyopathy is incompletely understood. While there is currently no evidence for a disease-causing role in the heterozygous state, compound heterozygosity of LOF variants along with missense variants can lead to extremely severe presentations, mimicking recessive inheritance (Hougs 2005, Girolami 2010). The CMP-EP assigned moderate weight to a LOF variant (PVS1_Moderate, Table 1), which yields a classification of VUS in the absence of case level data supporting pathogenicity.

Hougs L, Havndrup O, Bundgaard H, et al. One third of Danish hypertrophic cardiomyopathy patients with MYH7 mutations have mutations [corrected] in MYH7 rod region. Eur J Hum Genet. 2005;13(2):161-165.

Girolami F, Ho CY, Semsarian C, et al. Clinical features and outcome of hypertrophic cardiomyopathy associated with triple sarcomere protein gene mutations. J Am Coll Cardiol. 2010;55(14):1444-1453.

PM3 (variant detected in trans with a pathogenic variant): While compound heterozygosity leading to a more severe phenotype has been documented, this rule was designed for traditional recessive inheritance.

PP4 (proband's phenotype or family history is highly specific for a disease with a single genetic etiology): *MYH7*-associated disorders have high locus heterogeneity and cardiomyopathy can have non-genetic etiologies.

BS2 (observed in healthy adult with full penetrance expected at an early age): *MYH7*-associated diseases have reduced penetrance and can be adult-onset.

BP1 (missense variant in a gene where only LOF variant cause disease): *MYH7* LOF variants are exceedingly rare.

BP3 (in-frame deletions/insertions in a repetitive region without known functional domain): *MYH7* does not have repetitive regions.

PP5/BP6: reputable source reports variant as pathogenic/benign, but evidence is not accessible): The CMP-EP decided expert curations should only be used if accompanied by the evidence used. Platforms such as ClinVar enable laboratories to share the evidence on which an interpretation is based, and the CMP-EP encourages this practice.
