# Supplementary material for: Adaptation and validation of the ACMG/AMP variant classification framework for MYH7-associated inherited cardiomyopathies: recommendations by ClinGen’s Inherited Cardiomyopathy Expert Panel
Source: Genet Med. 2018 Jan 4;20(3):351–9. doi: 10.1038/gim.2017.218 (PMC5876064; doi:10.1038/gim.2017.218)
Supplement: Supplementary Text 3 [file gim2017218x5.pdf]

SUPPLEMENTARY TEXT 3

BA1 (MAF above which a variant can be classified as BENIGN assuming a MENDELIAN framework)

$$\left(\frac{[\text{disease prevalence}] \times [\text{gene contribution}]}{[\text{average variant penetrance}]} \right) \xrightarrow[\text{Poisson distribution}]{\text{95\% percentile of}} \geq 0.1\%$$

Assumptions (note: values deliberately set conservative to add “safety padding”)

- **Disease prevalence**: Hypertrophic cardiomyopathy is traditionally estimated to occur in 1/500 individuals[1] though recent estimates suggest that is may be higher[2]. Dilated cardiomyopathy was initially estimated to occur in 1 /2,500 individuals but like HCM is now estimated to be much more common[3]. Therefore, 1/200 individuals (1/400 chromosomes) was used as a “safe” value.
- **Penetrance**: Although *MYH7* is generally regarded as a “penetrant” cardiomyopathy gene, this is not well characterized at the variant level and therefore, to accommodate all variants, a penetrance value of 30% was used.
- **Gene contribution** was set at 10.6% based on the detection rate for HCM, which is the highest among *MYH7* associated cardiomyopathies[4].

BS1 (MAF too high for disease)

$$\left(\frac{[\text{disease prevalence}] \times \begin{matrix}[\text{maximum pathogenic} \\ \text{variant contribution}]\end{matrix}}{[\text{average variant penetrance}]} \right) \xrightarrow[\text{Poisson distribution}]{\text{95\% percentile of}} \geq 0.02\%$$

Assumptions

- **Disease prevalence:** 1/200 individuals (1/400 chromosomes)
- **Penetrance**: 30%
- **Maximum pathogenic variant contribution**: 2% based on *MYBPC3* variant p.Arg502Trp (Walsh et al. 2017[5]: 6,000 probands)
- **Note** that the FAF (95% poisson) is available for each variant in ExAC (http://exac.broadinstitute.org/).

REFERENCES:

1. Maron BJ, Spirito P, Roman MJ, et al. Prevalence of hypertrophic cardiomyopathy in a population-based sample of American Indians aged 51 to 77 years (the Strong Heart Study). *Am J Cardiol.* 2004;93(12):1510-1514.
2. Semsarian C, Ingles J, Maron MS, Maron BJ. New perspectives on the prevalence of hypertrophic cardiomyopathy. *J Am Coll Cardiol.* 2015;65(12):1249-1254.
3. Hershberger RE, Hedges DJ, Morales A. Dilated cardiomyopathy: the complexity of a diverse genetic architecture. *Nat Rev Cardiol.* 2013;10(9):531-547.
4. Alfares AA, Kelly MA, McDermott G, et al. Results of clinical genetic testing of 2,912 probands with hypertrophic cardiomyopathy: expanded panels offer limited additional sensitivity. *Genet Med.* 2015;17(11):880-888.
5. Walsh R, Thomson KL, Ware JS, et al. Reassessment of Mendelian gene pathogenicity using 7,855 cardiomyopathy cases and 60,706 reference samples. *Genet Med.* 2017;19(2):192-203.
