# Supplementary material for: Adaptation and validation of the ACMG/AMP variant classification framework for MYH7-associated inherited cardiomyopathies: recommendations by ClinGen’s Inherited Cardiomyopathy Expert Panel
Source: Genet Med. 2018 Jan 4;20(3):351–9. doi: 10.1038/gim.2017.218 (PMC5876064; doi:10.1038/gim.2017.218)
Supplement: Supplementary Appendix [file gim2017218x6.docx]

**Supplementary Appendix**

Banner for Authorship – ClinGen Cardiovascular Clinical Domain Working Group

Jonathan Berg, MD, PhD

Laura Milko, PhD

Andy Rivera

Michael Ackerman, MD, PhD

Melanie Care, Msc, CGC

Julie DeBacker, MD, PhD

Hal Deitz, MD

Michael Gollob, MD

Joshua Knowles, MD, PhD

Bart Loeys, MD

Bill McKenna, MD

Dianna Milewicz, MD, PhD

Valeria Novelli, PhD

Amy Sturm, MS, LGC

Arthur Wilde, MD, PhD

Banner Authorship, ClinGen, non-Cardiovascular Clinical Domain Working Group

Heidi Rehm, PhD

Euan Ashley, MD, PhD
